# Predictive Genomic Analyses Inform the Basis for Vitamin Metabolism and Provisioning in Bacteria-Arthropod Endosymbioses

**DOI:** 10.1534/g3.117.042184

**Published:** 2017-04-28

**Authors:** Laura R. Serbus, Brian Garcia Rodriguez, Zinat Sharmin, A. J. M. Zehadee Momtaz, Steen Christensen

**Affiliations:** * Department of Biological Sciences, Florida International University, Miami, Florida 33199; † Biomolecular Sciences Institute, Florida International University, Miami, Florida 33199

**Keywords:** symbiosis, endosymbiont, provisioning, vitamin, arthropod

## Abstract

The requirement of vitamins for core metabolic processes creates a unique set of pressures for arthropods subsisting on nutrient-limited diets. While endosymbiotic bacteria carried by arthropods have been widely implicated in vitamin provisioning, the underlying molecular mechanisms are not well understood. To address this issue, standardized predictive assessment of vitamin metabolism was performed in 50 endosymbionts of insects and arachnids. The results predicted that arthropod endosymbionts overall have little capacity for complete *de novo* biosynthesis of conventional or active vitamin forms. Partial biosynthesis pathways were commonly predicted, suggesting a substantial role in vitamin provisioning. Neither taxonomic relationships between host and symbiont, nor the mode of host-symbiont interaction were clear predictors of endosymbiont vitamin pathway capacity. Endosymbiont genome size and the synthetic capacity of nonsymbiont taxonomic relatives were more reliable predictors. We developed a new software application that also predicted that last-step conversion of intermediates into active vitamin forms may contribute further to vitamin biosynthesis by endosymbionts. Most instances of predicted vitamin conversion were paralleled by predictions of vitamin use. This is consistent with achievement of provisioning in some cases through upregulation of pathways that were retained for endosymbiont benefit. The predicted absence of other enzyme classes further suggests a baseline of vitamin requirement by the majority of endosymbionts, as well as some instances of putative mutualism. Adaptation of this workflow to analysis of other organisms and metabolic pathways will provide new routes for considering the molecular basis for symbiosis on a comprehensive scale.

Vitamins have long been recognized for their importance in biological systems. Ancient prescriptions of liver to treat night blindness, and use of citrus to prevent scurvy in the 1700s are now known to be effective because of their vitamin content (Whitney and Rolfes 2011). The 13 types of compounds conventionally recognized as vitamins (Table 1) broadly support central metabolism, the electron transport chain, signaling processes, and other functions. At the molecular level, vitamins assist in these functions through donation or acceptance of enzyme products, as well as by serving as coenzymes or prosthetic groups (Berg *et al.* 2002; Combs 2012). Thus, vitamins are essential to many biological processes. *De novo* vitamin biosynthesis requires the sequential action of many enzymes (Bender 2003), and thus retention of full synthetic capacity requires substantial energetic investment. Long-term exogenous availability of vitamins is expected to reduce selection for vitamin biosynthesis pathway genes (Shigenobu *et al.* 2000; Tamas *et al.* 2002; Gardner *et al.* 2002; Payne and Loomis 2006; Liu *et al.* 2006). The dietary vitamin requirements common to many organisms are consistent with that scenario.

**Table 1:** Vitamin nomenclature used in this study

Vitamin	Conventional Name	Compound Name	Active Form	KEGG Compound ID	Active Form Distinct from Vitamin Form
A	Retinol	*all*-*trans* Retinol	11-*cis* Retinal	C02110	+
B1	Thiamine	Thiamine	Thiamine diphosphate	C00068	+
B2	Riboflavin	Riboflavin	Flavin adenine dinucleotide (FAD)	C00016	+
			Flavin mononucleotide (FMN)	C00061	
B3	Niacin	Nicotinamide	Nicotinamide adenine dinucleotide (NAD)	C00003	+
		Nicotinate	Nicotinamide adenine dinucleotide phosphate (NADP)	C00006	
B5	Pantothenate	(*R*)-Pantothenate	Coenzyme A	C00010	+
B6	Pyridoxine	Pyridoxine	Pyridoxal-5-phosphate	C00018	+
B7	Biotin	Biotin	Biotin	C00120	−
B9	Folic acid	Folate	5,6,7,8-Tetrahydrofolate	C00101	+
B12	Cobalamin	B12a (commercial)	Adenosylcobalamin	C00194	+
		B12r (natural form)			
		B12s (natural form)			
C	Ascorbic acid	L-Ascorbate	L-Ascorbate	C00072	−
D	D2 or D3	Ergocalciferol	Ergocalciferol	C05441	−
		Cholecalciferol	Cholecalciferol	C05443	
E	Tocopherol	α-Tocopherol	α-Tocopherol	C02477	−
		α-Tocotrienol	α-Tocotrienol	C14153	
K	K1 or K2	Phylloquinone	Phylloquinone	C03313	−
		Menaquinone	Menaquinone	C00828	

Insects have been examined as a model for vitamin homeostasis for over nearly a century. The nutritionally limited diets of many insects inherently restrict their access to essential vitamins (Ziegler 1975; Sandström and Moran 1999; Bennett and Moran 2013; Loos-Frank and Lane 2017). The dietary availability of micronutrients in nature is also expected to vary in response to weather cycles, seasonal availability, and competition with other organisms. This raises questions about how insects acquire vitamins to meet their metabolic needs. Within the vast literature are classic examples of fruit fly dependency on vitamins B1, B2, B3, B5, and B6 for viability (Tatum 1939, 1941). Others have confirmed that fruit flies require vitamin B7 for creating fatty acid stores as well as for viability (Dadd 1973). Vitamin B9 has been demonstrated as required for successful larval/pupal development (Dadd 1973) as well as fecundity (Affleck *et al.* 2006). Dietary B12 stimulates insect growth and, in *Blattella germanica*, is required for egg viability (Nation 2015). Vitamin A is regarded as near universally required for insect vision (Dadd 1973; Nation 2015), and carotenoid precursors of vitamin A are important for aspects of cuticle pigmentation (Friend 1958; Dadd 1973). Vitamin C is recognized as a basic dietary requirement for many insects (Dadd 1973; Nation 2015) and has been highlighted as required for larval molting in *Manduca sexta* (Kramer and Seib 1982). Vitamin E was demonstrated as important for reproduction, with involvement in the fertilization, fertility, and fecundity of various insects (Dadd 1973). Growth impacts have also been associated with vitamins C and K (Dadd 1973; McFarlane 1978).

Mutualistic interactions between insects and endosymbiotic microbes that dwell within the body cavity have been widely discussed as a route to nutritional benefits (Buchner 1965; Moran and Baumann 2000; Dale and Moran 2006; Dale and Moran 2006). Up to 15% of insects are thought to carry such endosymbionts, referred to as primary if the relationship is ancient and obligate, and secondary if it is recent and facultative (Dale and Moran 2006; Moran *et al.* 2008; Wernegreen 2012). Provisioning roles for these endosymbionts have been primarily analyzed according to two strategies over the extended history of insect nutritional analyses. Classical tests generally have relied upon curing insects of their endosymbionts and testing for subsequent host micronutrient deficiencies by adding back vitamins in artificial media (examples reviewed in Buchner 1965; Douglas 1989; Heddi and Gross 2012). A collective limitation of these studies is that the distinction between vitamin contributions by endosymbionts and gut microbiota have not been systematically confirmed as separate. Recent work indicates that antibiotic treatments restructure the gut microbiome in insects (Gendrin *et al.* 2015; Ye *et al.* 2016; Raymann *et al.* 2017), as has also been seen in mammals (Antonopoulos *et al.* 2009; Jernberg *et al.* 2010; Buffie *et al.* 2012). The implications of these treatments are unclear, as the gut microbiome is implicated in vitamin provisioning (Tatum 1939; Friend 1958; Blatch *et al.* 2010; Piper *et al.* 2014; Wong *et al.* 2014; Nation 2015). The recent explosion of available endosymbiont genomes has provided a complementary route to exploring possible vitamin contributions by the endosymbionts (references in Supplemental Material, File S1 and Table S1). Each research group has individually confronted the shared challenge that metabolism is an extensive network of interactions that lack a single starting point. As there are no standard field definitions for what constitutes a functional vitamin biosynthesis pathway at this time, comparisons between studies are not currently possible.

To further consider the basis for vitamin provisioning by arthropod endosymbionts, it is necessary to address several open questions: (1) How do vitamin production strategies compare within and across endosymbiont taxonomic groups? (2) To what extent is vitamin biosynthesis capacity a product of endosymbiont-host relationships, endosymbiont genome size, or overall taxonomic limitations of endosymbiont capabilities? and (3) To what extent does endosymbiont vitamin production capacity align with endosymbiont vitamin requirements? This study addresses these questions through predictive analyses of vitamin biosynthetic capacity and vitamin dependency across endosymbiont taxa, as described below.

## Materials and Methods

### Selection of organisms

Endosymbiont selection was done in consultation with field literature and tailored to maximize diversity of endosymbiont representation. This study focused on bacterial endosymbionts that reside within the body cavity of insects and arachnids (Table S1). The genomes of all endosymbionts pursued here were confirmed as completely sequenced by NCBI, last accessed in March of 2017 (https://www.ncbi.nlm.nih.gov/genome/browse/). The organism list includes primary and secondary endosymbionts of a range of genome sizes (Table S1). Multiple strains of the same endosymbiont were pursued in some cases due to their prevalence in the current literature or the diversity of their associated host organisms. For selection of nonsymbiont taxonomic relatives, we focused on free-living organisms listed in the Superfamily 1.75 HHMI library and genome assignments server, last accessed in December of 2016 (http://supfam.org/SUPERFAMILY/), and the Kyoto Encyclopedia of Genes and Genomes (KEGG) database release 81.0, last accessed in February of 2017 (http://www.genome.jp/kegg/catalog/org_list.html). All nonsymbiont genomes were confirmed as complete by NCBI as above, last accessed in March of 2017. Taxonomic relationships of endosymbionts and nonsymbionts were confirmed by NCBI Taxonomy Tree, last accessed in November of 2016 (https://www.ncbi.nlm.nih.gov/taxonomy). Classical gut microbiota and specialized members of the gut microbiome that colonize gut crypts of insects (Kikuchi and Fukatsu 2014; Engel and Moran 2013) were not examined in this study.

### Vitamin pathway analysis

The KEGG database (Kanehisa and Goto 2000), which is based largely upon information from GenBank, was utilized as a major resource in the work. As of February of 2017, this database has been cited in >16,000 research articles. To confirm the accuracy of KEGG records related to vitamin metabolism, we tested for the presence of homologs of 39 vitamin-interacting proteins in 50 endosymbionts and 27 nonsymbiont relatives in both KEGG and NCBI Microbial BLAST, last accessed in January of 2017 (Table S1 and Table S2). This comparison ultimately excluded *Serratia symbiotica* from the study due to extensive disagreement between KEGG and BLAST entries displayed at that time. All other test queries returned identical results from both databases. Further information regarding specific Enzyme Commission numbers (ECs) was consulted in KEGG, NCBI, and PubMed whenever concerns arose about the pathway map displays. The EC predictions displayed within the pathway maps were confirmed as correct in all cases, except during an update of the entry 3.1.3.- to 3.1.3.104 in the riboflavin pathway that coincided with our use of the database. Lastly, it is not obvious from the KEGG (Kanehisa and Goto 2000; Kanehisa *et al.* 2016; Kanehisa *et al.* 2017) graphical display that EC 5.3.99.10 (*ten*I) is only required for thiamine biosynthesis in certain organisms (Hazra *et al.* 2011; Du *et al.* 2011; Begley *et al.* 2012).

To analyze vitamin biosynthesis pathways associated with endosymbionts and nonsymbiont relatives, a series of interconnected map diagrams was examined for each case (Table S3). Initial analysis determined that the input molecules standard to all pathways were amino acids, sugars, and nucleosidic bases (*i.e.*, guanine). Vitamin biosynthesis pathway branches identified as relevant to one or more endosymbionts during initial analysis were systematically analyzed in all organisms (Table S3). This ensured that any pathway branches originating from horizontal transfer would not accidentally be excluded from consideration. The outcome of this process was formalized in customized, adapted versions of the KEGG pathway maps, confirmed as suitable for this analysis, documented in Figure S1, Figure S2, Figure S3, Figure S4, Figure S5, Figure S6, Figure S7, Figure S8, Figure S9, Figure S10, Figure S11, and Figure S12 for reproducibility of the analysis by others. These maps were used as a guideline to analyze the predicted availability of enzyme homologs that carry out stepwise execution of the pathways. Pathway end points were set as either conventional or active vitamin forms (Table 1), depending upon the analysis.

**Figure 1:**
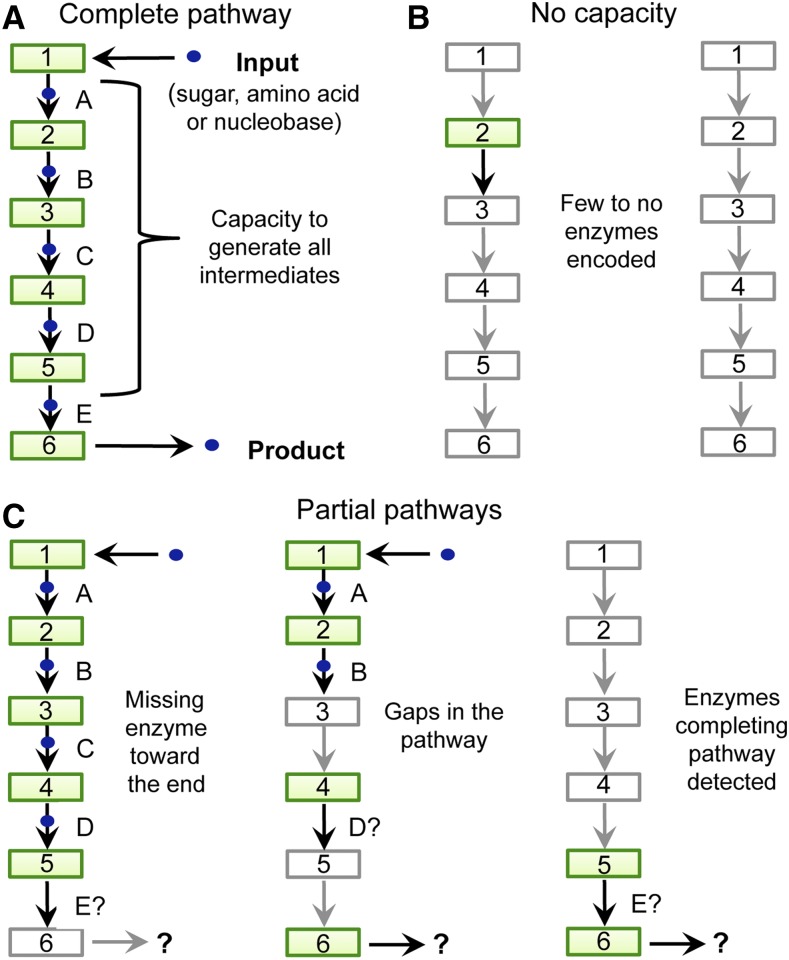
Pathway classification criteria. Circles: forms of the compound, ranging from Input, to intermediates (indicated by letters), to Final Product. Boxes: enzymes involved in pathway. Numbers indicate which step of the pathway is carried out by each enzyme. Green: predicted homolog of the enzyme has been reported. White: no enzyme homolog identified to date. (A) Representation of a complete pathway. (B) Pathways with no predicted synthetic capacity. (C) Partial pathways, synthetic capacity unclear.

Criteria were established to distinguish between predicted complete, partial and nonfunctional pathways (Figure 1). Several entries were also predicted as complete after considering findings from the literature. Unlike other types of bacteria, thiamine biosynthesis in *Escherichia coli* does not require EC 5.3.99.10 (Du *et al.* 2011). A small set of thiamine pathways from other organisms were assumed complete because all enzymes necessary to complete the pathway were predicted as available except for EC 5.3.99.10. A subset of other pathways was classified as complete based on predicted availability of *ilv*C and *gap*A (Table S4), which have respectively been identified as functional substitutes for EC 1.1.1.169 in the pantothenate pathway (Price and Wilson 2014) and for EC 1.2.1.72 in the riboflavin pathway (Yang *et al.* 1998; Michalkova *et al.* 2014).

### Development and application of the DataMiner application

To systematically identify associations between predicted enzyme homologs and the 77 organisms of interest in this study, a Java application referred to as DataMiner was developed using NetBeans IDE 8.1 and Java SE runtime environment 1.8.0, completed in December of 2016. The application is expected to work on all operating systems (including Apple, Windows, and Linux) that have an appropriate Java runtime environment installed. The DataMiner program code is provided in File S2 and File S3. Lists of enzymes can be cross-referenced against our organisms of interest in the Java-based DataMiner application (File S4). All DataMiner resources are also available for public download from http://faculty.fiu.edu/∼lserbus/Serbus_Lab/Research.html

The DataMiner application begins by reading a user-generated list of EC numbers from a .txt file, formatted to show one EC identifier per line. Afterward, DataMiner accesses the online KEGG enzyme database sequentially for each EC through a URL that includes the active EC number (http://www.genome.jp/dbget-bin/www_bget?ec:<EC Number>), then generates a list of all organisms reported to use this enzyme class. In the KEGG enzyme database, organisms are cataloged through an all-capital abbreviation, usually ranging from three to four letters (*e.g.*, DME is the abbreviation for *Drosophila melanogaster*). Once the organism list is collected for a given enzyme class, DataMiner compares the list to a separate, predefined list of organisms of interest. If an enzyme class is used by an organism of interest, as indicated by the presence of the organism abbreviation in the online organism list, DataMiner reports a 1 for the EC number of that organism. Negative results are reported as a 0. The process is reiterated for each EC number in the list until it reaches the end and an output text file is created and placed on the desktop. The format of the output text file is written for compatibility with import into Excel, using spaces as delimiters.

In addition to the core DataMiner functions, a few subroutines are included in the application to prevent errors that may arise with obsolete or deleted EC numbers, or EC numbers unique to a single organism which lack an organism list. In the KEGG database, obsolete EC numbers are usually accompanied with a link to the new EC number representing the obsolete enzyme class. DataMiner is capable of redirecting itself to the new EC number when it encounters an obsolete number. When this occurs, DataMiner reports the obsolete EC number to the user interface and replaces it with the new one so long as the new EC number is not already present in the given EC number list. If DataMiner is unable to find an organism list because the EC number lacks a specific organisms list or because the EC number entry has been deleted, DataMiner does not include that EC number into the exported file and reports the absence of the organism list to the user interface.

Controlled scenarios posing ideal conditions and potential pathway blocks were presented to the DataMiner application in order to assess its efficacy. In ideal conditions, no EC numbers are obsolete or missing organism lists. A smaller subset of an EC list containing only ideal EC numbers was run in parallel through the Java version of DataMiner (File S4) and an Excel-based version of DataMiner (File S5) to confirm that both were operating appropriately. This test confirmed that both versions of DataMiner operated with equivalent accuracy. Nonideal factors, such as obsolete ECs and ECs with missing organism lists, were next introduced to determine whether DataMiner’s automated output for reported ECs would change. The results yielded by nonideal and ideal datasets were determined to be identical.

To predict expected vitamin usage by organisms of interest, a raw list of EC numbers representing enzymes that interact with activated vitamin forms was compiled. For the majority of cases, the IDs of vitamin-utilizing enzymes were sourced from the EMBL-EBI CoFactor database v2.1.1, last accessed in April of 2017 (http://www.ebi.ac.uk/thornton-srv/databases/CoFactor/index.php). Each activated vitamin form was located in the “Browse cofactors” menu, and lists of vitamin-interacting enzymes located under the “enzymes and domains” header. EC numbers were selected from the lists “Uses vitamin as a cofactor” and “Vitamin-binding.”

To predict the ability of an endosymbiont to generate active vitamins through last-step conversion of intermediate compounds, a list of EC numbers representing enzymes that directly generate each active form was compiled. The final content of these lists is based on visual inspection of KEGG pathway maps (Figure S1, Figure S2, Figure S3, Figure S4, Figure S5, Figure S6, Figure S7, Figure S8, Figure S9, Figure S10, Figure S11, Figure S12, and Table S3), cross-referenced with the vitamin-synthesizing enzyme lists provided for each vitamin by EMBL-EBI CoFactor. Most enzyme lists were then run through the Java version of DataMiner (File S4) to identify homologs in organisms of interest. Short enzyme lists were manually compared against organisms of interest using the Excel-based version of DataMiner (File S5).

### Statistical analyses

Regression analysis was performed in IBM SPSS Statistics, version 23, using the Curve Estimation function. Chi-squared tests and *post hoc* analyses were also performed using SPSS as previously described (Beasley and Schumacker 1995).

### Data availability

The authors state that all data necessary for confirming the conclusions presented in the article are represented fully within the article.

## Results

### Endosymbionts have little predicted capacity for complete de novo biosynthesis of conventionally recognized vitamins

To elucidate the mechanisms of vitamin provisioning by endosymbionts, the capacity of endosymbionts for vitamin biosynthesis was initially investigated. This was first pursued through analysis of vitamin pathway predictions from 50 sequenced bacterial endosymbionts of insect and arachnids (Table S1). The endosymbiont selection was tailored to maximize taxonomic diversity of the endosymbionts and their hosts. For each endosymbiont, networks of KEGG pathway maps associated with vitamin biosynthesis were examined. In practice, this involved working backwards from final vitamin products through all branches of metabolism that potentially contribute to synthesis of the product. Consensus starting molecules were amino acids, sugars, or nucleosidic bases (Figure S1, Figure S2, Figure S3, Figure S4, Figure S5, Figure S6, Figure S7, Figure S8, Figure S9, Figure S10, Figure S11, Figure S12, and Table S3). Predicted pathway activities that support biosynthesis of all 13 conventionally recognized vitamins were analyzed (Table S3). There is no evidence to date that eubacteria synthesize vitamin E (Mokrosnop 2014), and according to KEGG, only 0.2% of sequenced bacteria are predicted to have partial capacity for synthesizing vitamin D. As such, vitamin D and vitamin E pathways served as negative controls and will not be discussed further.

**Figure 2:**
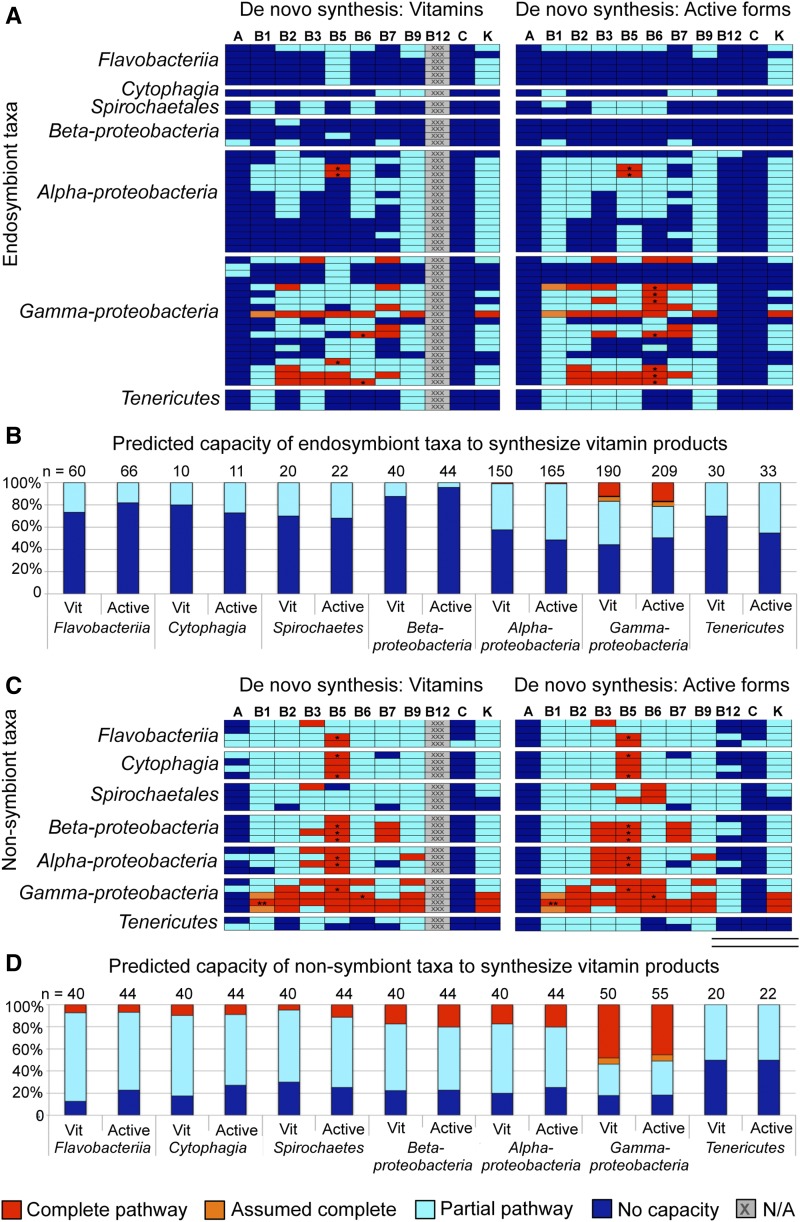
Predictions of vitamin pathway capacity in arthropod endosymbionts and nonsymbiont taxonomic relatives. Red: pathways predicted as complete. Orange: pathway assumed complete. Cyan: predicted as a partial pathway. Dark blue: no predicted synthetic capacity. *Prediction includes contribution of a substitute enzyme. **Pathway has been confirmed as functional *in vitro*. (A) Left side: endosymbiont capacity for *de novo* biosynthesis of conventional vitamin forms. Right side: capacity for *de novo* biosynthesis of active vitamin forms. (B) Overall synthetic capacity of each taxonomic group for conventional *vs.* active vitamin biosynthesis. (C) Left side: nonsymbiont capacity for *de novo* biosynthesis of conventional vitamin forms. Right side: capacity for *de novo* biosynthesis of active vitamin forms. (D) Summary: synthetic capacity of each taxonomic group for conventional *vs.* active vitamin biosynthesis.

To predict the capacity of endosymbionts for complete *de novo* vitamin biosynthesis, pathways were examined for coding predictions of all enzymes involved in stepwise conversion of starting molecules into the final product. According to this analysis method, complete and assumed complete *de novo* vitamin biosynthesis was predicted to be possible in 27 pathways of 12 endosymbionts, representing 5% of pathways overall (*n* = 500 pathways of 50 endosymbionts) (Figure 1A, Figure 2A, and Table S5). An additional 36% of pathways analyzed overall were predicted as having partial capacity for vitamin biosynthesis (Figure 1C and Figure 2, A and B). The remaining 59% of pathways were predicted to exhibit no capacity for vitamin biosynthesis according to the analysis criteria (*n* = 500) (Figure 1B, Figure 2, A and B, and Table S5). The γ-proteobacteria predictions stood out, with significantly more pathways classified as complete (*P* < 0.0001, adjusted α = 0.0024) and significantly fewer pathways with no apparent capacity for vitamin biosynthesis (*P* < 0.00021, adjusted α = 0.0024, *n* = 500) as compared to all other endosymbiont taxa. However, the data overall predict that endosymbionts of arthropods have little capacity for complete *de novo* biosynthesis of conventional vitamins. The implication of the currently available data are that complete *de novo* biosynthesis of vitamins does not serve as the foundation for vitamin provisioning by bacterial endosymbionts of arthropods.

### Predicted endosymbiont pathway capacity for de novo biosynthesis of active vitamin forms is similar to that of conventional vitamins

It is known that the majority of vitamins must be converted to structurally distinct, active forms to function (Table 1). Some active forms can be synthesized independently of the conventional vitamin altogether (Figure S2, Figure S5, Figure S6, Figure S7, Figure S9, and Figure S10). To predict endosymbiont synthetic capacity from the perspective of active vitamin forms, the vitamin pathway analysis was repeated with alternate end points, when appropriate (Figure S1, Figure S2, Figure S3, Figure S4, Figure S5, Figure S6, Figure S7, Figure S8, Figure S9, Figure S10, Figure S11, Figure S12, and Table S3). This analysis predicted 37 pathways as capable of either complete or assumed complete *de novo* biosynthesis of active vitamin forms, representing 7% of all pathways analyzed (*n* = 550 pathways of 50 endosymbionts) (Figure 1, Figure 2A, and Table S6). Partial synthetic capacity was predicted for 35% of pathways analyzed (Figure 2, A and B). Similar to above, the γ-proteobacteria were predicted to exhibit significantly more complete pathways than other taxa (*P* < 0.0001, adjusted α = 0.0024, *n* = 550 pathways in seven taxonomic classes) (Figure 2, A and B). With respect to the active vitamin forms synthesized, predicted pathway functions were attributable to vitamins B1–B9 and vitamin K synthesis (Figure S13A). The other 58% of pathways were predicted as having no functional capacity according to analysis of current data (*n* = 550 pathways of 50 endosymbionts) (Figure 1, Figure, 2 A and B, and Table S6). Overall, predicted endosymbiont capacity for synthesizing active vitamin forms was not significantly different from conventional vitamins (Figure 2B). According to these predictions, complete *de novo* synthesis does not provide the foundation for provisioning of active vitamin forms. The higher frequency of predicted partial pathways is consistent with an expectation that the enzymes are still used (Andersson and Andersson 1999). Perhaps partial pathways contribute overall to vitamin biosynthesis through conversion of otherwise available intermediates into active vitamin forms.

### Endosymbiont-host relationships are not clear predictors of endosymbiont pathway capacity for vitamin biosynthesis

As less than half of endosymbiont vitamin biosynthesis pathways were predicted to be functional, this raises questions about which factors are associated with retention of this capacity. One possibility is that different host taxa have differentially selected for retention of endosymbiont vitamin synthetic capacity. Clustering the endosymbiont pathways by host taxonomic order did indicate that endosymbionts of Hemiptera exhibited less predicted synthetic capacity than the endosymbionts associated with other host taxa (*P* < 0.0001) (Figure S14B). However, substantial asymmetry in the number and diversity of endosymbiont taxa associated with each host order in currently available data limits the implications of this result (Figure S14A). Similar issues arose during assessment of synthetic capacity in primary *vs.* secondary endosymbionts. The data suggested that primary endosymbionts have significantly less predicted capacity than secondary endosymbionts for biosynthesis of active vitamin forms (*P* < 0.0001) (Figure S14C). However, only α- and γ-proteobacteria were represented in both primary and secondary endosymbiont categories, inherently limiting interpretation of the analysis. Thus, currently available data do not predict a clear overall association of host type or host-symbiont relationship with retention of endosymbiont vitamin pathway capacity.

### The protein-coding capacity of endosymbionts correlates moderately with their predicted capacity to synthesize active vitamin forms

**Figure 3:**
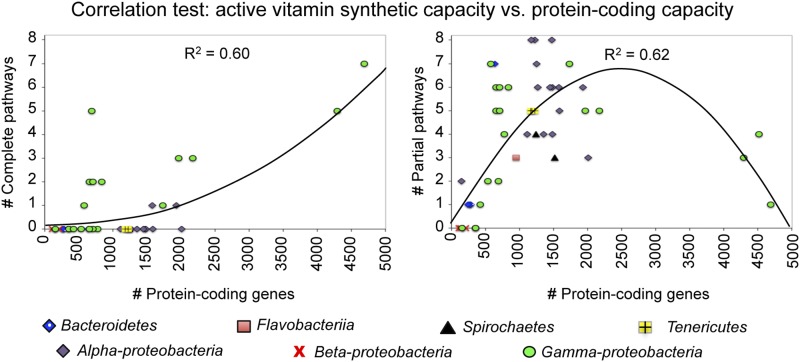
Regression analysis to test the relationship between vitamin biosynthesis predictions and endosymbiont protein-coding capacity. Best-fit models were determined by SPSS. Taxonomic associations of each data point are indicated by symbols explained below the graphs. Left panel: for each endosymbiont, predictions of complete vitamin biosynthesis pathways were compared against the number of protein-coding genes. Right panel: comparison of partial vitamin pathways against the number of protein-coding genes for each endosymbiont.

Endosymbiont genomes are thought to undergo reduction over time (Mira *et al.* 2001; Moran 2002), thus a sensible expectation would be for the extended pathways of vitamin metabolism to be affected. To test for a correlation between vitamin biosynthetic capacity (Figure 2A) and overall protein-coding capacity (Table S1) of the endosymbionts, a regression analysis was performed. The relationship between predicted *de novo* biosynthesis of activated vitamin forms and the protein-coding capacity of each organism was analyzed (Figure 3). The best-fit model for pathways predicted as complete and assumed complete showed increasing pathway capacity as a function of larger genome sizes (Figure 3, left panel). The best-fit model for pathways with predicted partial synthetic capacity showed initial increases in pathway capacity for smaller genome sizes that plateaued and ultimately decreased for organisms with larger genome sizes (Figure 3, right panel). These trends comply with an expectation of tradeoffs between finite numbers of complete and incomplete pathways in organisms with differential synthetic competency. As the models accounted for 60–62% of the data (Figure 3), the implication is that genome size could be one of several factors contributing to predicted vitamin biosynthetic capacity of endosymbionts.

### The predicted capacity of endosymbiont vitamin biosynthesis pathways in part reflects broader metabolic limitations of their taxa

Another possibility is that the vitamin metabolic capacity of endosymbionts reflects generalized synthetic limitations of their taxa. To address this possibility, vitamin biosynthesis pathways were analyzed in 27 sequenced nonsymbiont taxonomic relatives of the endosymbionts (Figure S1, Figure S2, Figure S3, Figure S4, Figure S5, Figure S6, Figure S7, Figure S8, Figure S9, Figure S10, Figure S11, Figure S12, Table S1, and Table S3). A total of 49 pathways from 27 nonsymbionts were predicted to be capable of complete *de novo* vitamin biosynthesis (Figure 1A, Figure 2C, and Table S7). Including two pathways that were assumed complete, this synthetic capacity is predicted for 19% of pathways analyzed (*n* = 270). Partial pathways were predicted for 59% of pathways analyzed (*n* = 270) (Figure 1C and Figure 2, C and D). The remaining 22% of pathways were classified as nonfunctional (*n* = 270) (Figure 1B, Figure 2, C and D, and Table S7). Overall, the γ-proteobacteria stood out from the other taxa, with significantly more functional pathways and significantly fewer partial pathways (*P* < 0.0001, adjusted α = 0.042, *n* = 270). Compared against the endosymbiont data, the nonsymbiont relatives were predicted to have significantly more vitamin biosynthetic capacity (*P* < 0.0001), with significant differences observed in all taxonomic groupings analyzed except Tenericutes (Table S8). Thus, predicted capacity for vitamin biosynthesis was significantly higher in the nonsymbiotic bacteria that were analyzed than for most taxonomically related endosymbionts.

Analysis of pathways supporting *de novo* biosynthesis of active vitamin forms yielded similar results (Figure S1, Figure S2, Figure S3, Figure S4, Figure S5, Figure S6, Figure S7, Figure S8, Figure S9, Figure S10, Figure S11, Figure S12, and Table S3). A total of 55 pathways were predicted to have complete biosynthetic capacity, and three additional pathways were assumed to meet this standard, together representing 20% of pathways analyzed (*n* = 297) (Figure 1A, Figure 2C, and Table S9). Further, 55% of pathways were classified as capable of partial biosynthesis of active vitamin forms (Figure 2, C and D). Predicted synthetic capacity was associated with production of all B vitamins, vitamin K, and to a minor extent, vitamin C (Figure S13B). Finally, 25% of pathways were predicted to have no capacity for biosynthesis of active vitamin forms (Figure 2, C and D). As before, γ-proteobacteria pathways showed significantly more predicted capacity for complete biosynthesis (*P* < 0.0001, adjusted α = 0.0042) and significantly fewer nonfunctional pathways than other taxa (*P* < 0.0017, adjusted α = 0.0042, *n* = 297) (Figure 2, C and D). Comparisons between endosymbionts and nonsymbionts within shared taxonomic groupings were predicted to show significant differences in all cases except Tenericutes (Table S8). This predictive data suggests that the endosymbionts carry a reduced form of the limited synthetic capacity endogenous to nonsymbiont relatives.

### Endosymbionts are predicted to have substantial capacity for last-step conversion of intermediate compounds into active vitamin forms

As partial synthetic pathways were predicted more commonly than complete ones in arthropod endosymbionts, one possibility is that vitamin pathway fragments have utility because the intermediates they generate have important roles. Support was detected for synthesis of provitamin A forms like lycopene, β-carotene, and astaxanthin, as well as the cob(II)alamin form of B12 (Table S10), which are associated with antioxidant roles in other systems (Di Mascio *et al.* 1989; Naguib 2000; Birch *et al.* 2009; Suarez-Moreira *et al.* 2009; Milani *et al.* 2016). It is also possible that partial vitamin pathways carry out an important role in vitamin biosynthesis by simply processing available molecular intermediates into active vitamin forms. This type of capacity for vitamin biosynthesis has not been systematically investigated in the context of endosymbionts or their relatives to date.

**Figure 4:**
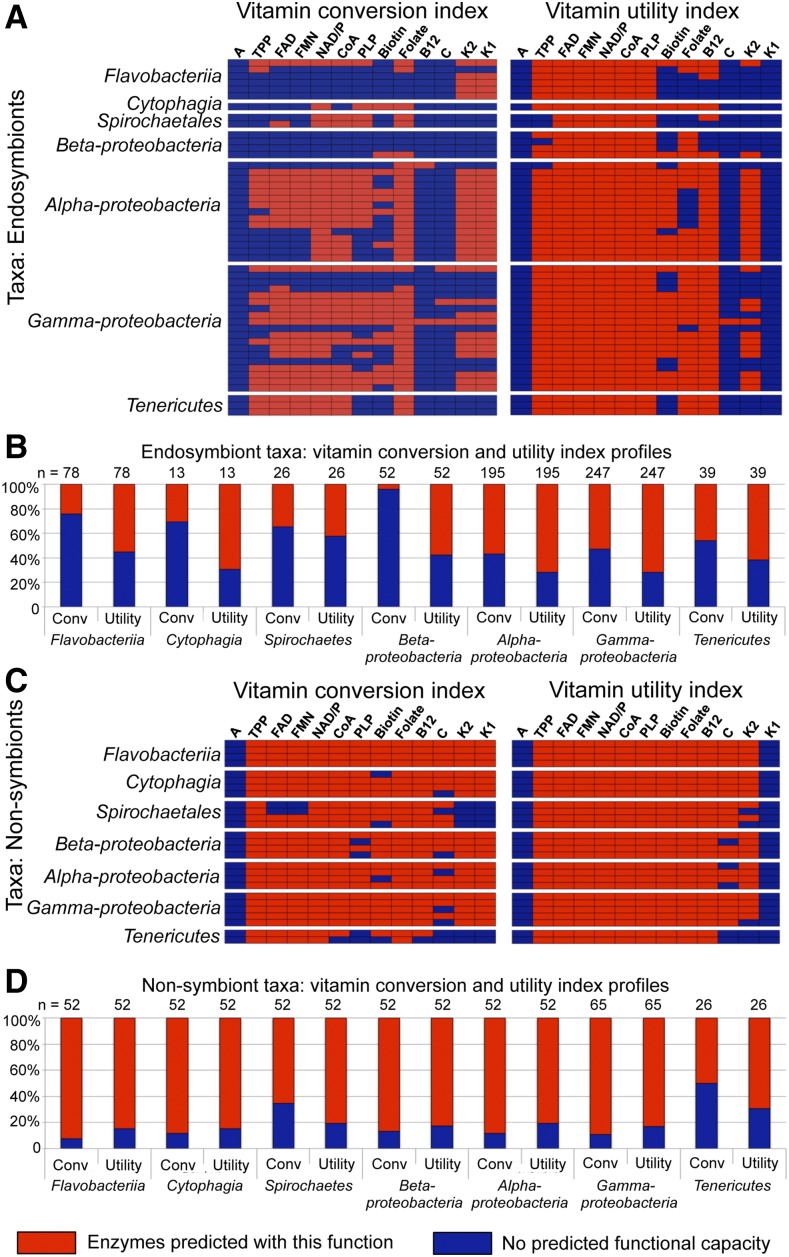
Software predictions of vitamin conversion and vitamin utility index across endosymbiont and nonsymbiont taxa. Red: organism has one or more predicted enzyme homologs that are directly relevant to the vitamin examined. Blue: organism had no predicted homologs of the related enzymes. (A and B) Endosymbiont predictions. (A) Predicted capacity to (left panel) convert intermediates directly into active vitamin forms and (right panel) utilize active vitamin forms. (B) Overall comparisons of vitamin conversion and utility by endosymbiont taxonomic groups. (C and D) Predictions for nonsymbionts that are taxonomically related to arthropod endosymbionts. (C) Predicted capacity to (left panel) convert intermediates directly into active vitamin forms and (right panel) utilize active vitamin forms. (D) Overall comparisons of vitamin conversion and utility by nonsymbiont taxonomic groups.

To comprehensively predict endosymbiont ability to synthesize active vitamin forms, we compiled a list of enzymes with the capability to convert an intermediate compound directly into an active vitamin form. We then created a computer program to locate lists of organisms encoding each enzyme and compare those against our organisms of interest (Figure S15 and File S6). These data were used to predict availability of enzymes in each endosymbiont that could generate each active vitamin form (Figure 4A and Table S11). This analysis predicted that endosymbionts have capacity for last-step conversion of intermediates into active vitamin forms in 45% of cases examined overall (*n* = 650). The Flavobacteriia and β-proteobacteria endosymbionts were predicted to have significantly lower capacity for vitamin conversion than other endosymbionts (*P* = 0.00010 and *P* < 0.00001, respectively; adjusted α = 0.00357). Conversely, α- and γ-proteobacterial endosymbionts were predicted to have significantly higher capacity for vitamin conversion (*P* = 0.00006 and *P* = 0.00270, respectively; adjusted α = 0.00357). These data predict that routes for last-step conversion of intermediates into active vitamin forms are differentially available across taxonomic groups of endosymbionts.

### Endosymbionts are predicted to widely utilize active vitamin forms

As vitamins support core metabolic processes, endosymbionts are expected to also have some extent of vitamin requirement to sustain basic functions before any provisioning role is carried out. To predict endosymbiont vitamin usage, we compiled a list of vitamin-utilizing enzymes and applied the computer program we developed to predict which endosymbionts encode these enzymes (Figure S15 and File S7). The data are presented in terms of a vitamin utility index (Figure 4A and Table S12). Analysis of the vitamin utility index for the endosymbionts predicted vitamin utility in 67% of the cases analyzed (Figure 4A) (*n* = 650). Comparison of the vitamin conversion index to the vitamin utility index at the level of endosymbiont taxonomic groups predicted that vitamin utility consistently exceeds the capacity for vitamin conversion overall (Figure 4, A and B). Overall, these data suggest that endosymbionts require exogenous supplementation of at least some critical vitamins.

### Endosymbiont dependency on exogenous vitamins is a generalized property of their taxa

To determine whether predicted endosymbiont vitamin dependencies are specific or represent generalized limitations of their taxa, nonsymbiont taxonomic relatives were also analyzed. Analysis of nonsymbionts predicted capacity to convert intermediates into active vitamin forms in 83% of cases examined (*n* = 351) (Figure 4C, Table S11, and File S6). This predicted capacity for vitamin conversion was significantly higher for nonsymbionts than endosymbionts in all taxonomic groupings except Tenericutes (Table S8). This suggests that nonsymbionts generally have more options for production of active vitamin forms than endosymbionts, even at the level of last-step conversion of intermediates.

Assessment of vitamin utility by nonsymbionts also predicted vitamin use by these organisms in 81% of cases examined (*n* = 351) (Figure 4C, Table S12, and File S7). For Spirochaetales and Tenericutes, the data predicted that vitamin utility exceeds the vitamin conversion capacity of these organisms. This parallels the patterns exhibited by endosymbionts of the same taxonomic groups (Figure 4, B and D). For all other nonsymbiont taxa analyzed, vitamin use was more than amply covered by their predicted vitamin conversion abilities (Figure 4D). This contrasts with the vitamin dependencies predicted for endosymbionts of the same taxonomic groups (Figure 4B) and suggests that a loss of vitamin conversion capacity has occurred in the endosymbionts. It is also noteworthy that vitamin utility was predicted as not significantly different between endosymbionts and nonsymbionts of the taxa Cytophagia, α-proteobacteria, γ-proteobacteria, and Tenericutes (Table S8). This implies that loss of vitamin conversion abilities has preceded loss of vitamin use in several endosymbiont lineages.

### Endosymbiont vitamin conversion and utility are predicted to parallel one another overall

**Figure 5:**
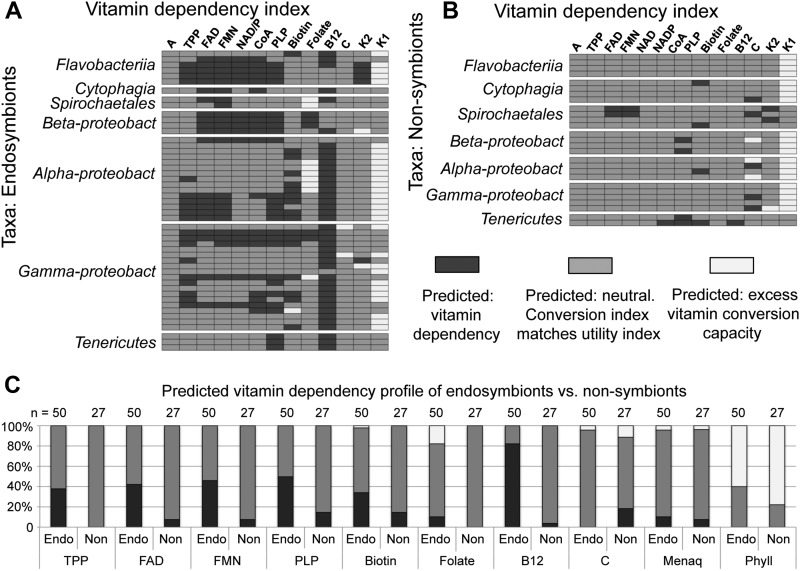
Predicted vitamin dependency index of endosymbionts and related nonsymbionts. The images show case-by-case interpretations from comparison of the vitamin conversion index to the vitamin utility index. Light gray: cases where last-step conversion of an intermediate into an active vitamin form was predicted, but no utility for the product was apparent. Medium gray: cases where capacity was predicted for both vitamin conversion and vitamin utility by the organism. Dark gray: cases where no capacity for vitamin conversion was detected, though utility of the active vitamin product was predicted. Comparisons of the predicted vitamin utility index against the predicted vitamin conversion index are displayed for (A) endosymbionts and (B) non-symbiont taxonomic relatives of the endosymbionts. (C) The predicted dependency profiles for endosymbionts versus non-symbionts are also displayed with respect to each active vitamin form.

To further examine the extent of exogenous vitamin dependency, the vitamin conversion index and vitamin utility index predictions from endosymbionts were directly compared, resulting in a vitamin dependency index (Figure 5). In 65% of cases examined, capacity for both conversion and utility of the same vitamin was predicted, consistent with the possibility of matched capacity (*n* = 650) (Figure 5A and Table S13). In 29% of cases, the utility of a given vitamin was predicted to exceed the capacity of the organism to synthesize it, invoking a putative dependency (*n* = 650). This outcome was associated to some extent with all endosymbiont taxa, most predominantly active vitamin B12 (*n* = 41 out of 50 cases). Other capacity mismatches suggestive of dependency were associated with vitamins B6 (25 cases), B2 (FAD, 21 cases; FMN, 23 cases), and B1 (19 cases) (*n* = 50 each) (Figure 5A). Comparison of the vitamin conversion and utility indices of each endosymbiont additionally highlighted 7% of cases in which apparently unnecessary synthetic ability was predicted (*n* = 650). This was most commonly associated with vitamin K1 (30 cases), and to a lesser extent, folate (nine cases), ascorbate (two cases), vitamin K2 (two cases), and biotin (one case) (*n* = 50 each) (Figure 5A). In the absence of other information, apparently unnecessary capacity for vitamin biosynthesis is consistent with the possibility of vitamin provisioning by these endosymbionts.

Addressing the extent to which vitamin dependency outcomes correlate with endosymbiosis, the vitamin dependency index of nonsymbiont relatives was also examined. This analysis indicated that in 87% of cases, predictions of synthetic capacity were matched with predictions of vitamin utility by the organism (*n* = 351) (Figure 5B and Table S13). In 7% of cases, vitamin utility by the organism was predicted to exceed capacity to convert the necessary intermediates (*n* = 351). As 11 out of 21 instances of apparent vitamin dependency predicted in nonsymbionts did not parallel the endosymbiont profile (Figure 5C), the data imply that substantial deficiencies in vitamin production for both endosymbionts and nonsymbionts have arisen since divergence. Though no cases of apparently unnecessary synthetic capacity were expected from nonsymbionts, the analysis did also predict this in 6% of cases, specifically for vitamin K1 (21 cases) and vitamin C (three cases) (Figure 5B). As these outcomes stand out for both endosymbionts and nonsymbionts in the comparative data, further consideration is likely appropriate.

## Discussion

In order to understand the basis for vitamin provisioning, this study comprehensively addressed the predicted capacity of endosymbionts for vitamin production. By applying systematic methods, broad comparative assessments across pathways and organisms were performed. The strictest method of predicting biosynthetic capacity was to assess vitamin biosynthesis pathways between comparable start and end points. The strengths of this analysis are the straightforward frame of reference for comparison of pathways and overlap with the scope of prior analyses of vitamin metabolism (Heddi *et al.* 1999; Nakabachi and Ishikawa 1999; Dunning Hotopp *et al.* 2006; Wu *et al.* 2006; Degnan *et al.* 2009; McCutcheon *et al.* 2009; Sabree *et al.* 2009; Kirkness *et al.* 2010; Snyder *et al.* 2010; Lamelas *et al.* 2011; Penz *et al.* 2012; Siddaramappa *et al.* 2012; Husnik *et al.* 2013; Lopez-Madrigal *et al.* 2013; Nakabachi *et al.* 2013; Koga and Moran 2014; Price and Wilson 2014; Nikoh *et al.* 2014; Wulff *et al.* 2014; Moriyama *et al.* 2015; Smith *et al.* 2015; Williams and Wernegreen 2015; Novakova *et al.* 2016). The most expansive method of predicting vitamin biosynthetic capacity was to assess last-step conversion of intermediates into active vitamin forms. The strength of this approach is that it accounts for the possible contribution of intermediates produced by conventional vitamin pathway fragments as well as salvage pathways. This study did not attempt to reconcile possible sources of these intermediates, which would have to be made available by coresident endosymbionts and/or the host organism. Though thoughtful studies have presented evidence for interpathway genomic complementation in endosymbiosis [reviewed in Mori *et al.* (2016)], the availability of endosymbiont transporters to support exchange of such intermediates appears to be limited (Zientz *et al.* 2004; Charles *et al.* 2011). It is not currently known how permissive these endosymbiont transporters are overall, nor is it known to what extent critical intermediates are exchanged by diffusion through lipid bilayers (Mori *et al.* 2016). Addressing feasibility and modes of intermediate exchange will require future experiments.

The two methods of analyzing vitamin biosynthesis provide complementary insights into strategies employed by endosymbionts. The pathway analysis method predicted complete *de novo* biosynthetic capacity for a subset of vitamin pathways in the γ-proteobacteria, and not in most other endosymbiont taxa, suggesting that this capacity is rare among arthropod endosymbionts overall. It is possible that the insights yielded by extensive study of culturable γ-proteobacteria, like the model organism *E. coli* (Blount 2015), do not fully encompass the strategic variations in vitamin biosynthesis employed by other taxa. However, predictions based on current, publicly available data indicate that the endosymbionts of insects and arachnids overall encode far more partial than complete pathways. This opens the possibility that endosymbionts regularly use partial pathways to synthesize active vitamin forms.

One goal of this work was to address the basis for differences in vitamin biosynthesis capacity of arthropod endosymbionts. Some association between predicted pathway capacity and genome size was indicated by the data, consistent with broad expectations from genome size reduction in endosymbionts (Moran and Bennett 2014). The predictive data also indicated that limited vitamin pathway capacity in endosymbionts partially reflected the overall synthetic capabilities of their taxa. Ultimately, the best overall predictor of vitamin synthetic capacity was the extent of vitamin use by each organism. A total of 249 out of 293 cases of predicted vitamin conversion capacity were paralleled by predictions of vitamin use by the endosymbiont. This extensive overlap implies that vitamin biosynthetic capacity in endosymbionts is retained, in large part, for the benefit of the endosymbiont, with provisioning being a secondary feature.

The comparative data provide a frame of reference for further study considering the basis for vitamin provisioning. Though this analysis predicted that *Wigglesworthia* has the capacity to both produce and utilize active vitamins B1, B6, and B9, it has also been demonstrated that *Wigglesworthia* provisions these same vitamins to the host and the coresident endosymbiont *Sodalis glossinidius* (Snyder *et al.* 2010; Snyder *et al.* 2012; Michalkova *et al.* 2014; Snyder and Rio 2015). Similarly, this study predicted capacity for both production and utilization of active vitamin B12 by *Hodgkinia*, though genomic evidence in the literature also supports a provisioning role (McCutcheon *et al.* 2009). Taken together, these data suggest that any endosymbionts in this study with displayed predictions of matched capacity for vitamin synthesis and utility could potentially upregulate vitamin production for provisioning purposes (Moran 2006). The recent demonstration of highly abundant biosynthetic enzymes in the proteome of *Blochmannia* (Fan *et al.* 2013) is consistent with expectations for overproduction as a nutritional provisioning strategy.

The absence of enzyme classes in some taxa also opens the possibility of alternate modes of provisioning. Cases of apparently unnecessary capacity for last-step conversion were associated with five vitamins across the 34 endosymbionts and 21 nonsymbionts analyzed. Empirical experiments would be needed to confirm whether endosymbiont homologs of the enzymes driving last-step vitamin conversion are, in fact, able to produce these products. In the event that synthesis is achieved, it is possible that these products serve an alternate function. For example, phylloquinone (K1) can reportedly serve as a transcriptional regulator (Combs and McClung 2017) and is capable of sequestering high-energy electrons (Brettel *et al.* 1987; Petersen *et al.* 1987), an overall effective strategy for suppression of reactive oxygen (Li *et al.* 2003). Alternatively, it could be speculated that arthropod hosts may gain other selective advantages by acquiring such products through endosymbiont provisioning.

The data presented by these analytical methods also provide a new perspective from which to consider the basis of mutualism in vitamin metabolism. Some extent of endosymbiont dependency upon the host is intrinsic to many endosymbiotic relationships (Douglas 1989; Moran 2006; Dale and Moran 2006). The predicted absence of enzyme classes invokes a possible requirement for exogenous supply of specific vitamin products. For example, this study highlights *Candidatus* *Portiera aleyrodidarum* as one of the costliest endosymbionts with respect to vitamin metabolism, with predicted use of eight active vitamin forms that the organism has no predicted capacity to generate. Tolerance of this predicted cost by the host further highlights the importance of endosymbiont-provisioned amino acids and carotenoids that the host reportedly receives as a trade-off (Santos-Garcia *et al.* 2012). Another possibility that cannot be ruled out is that accumulated mutations (Moran 1996; Wernegreen and Moran 1999; Fares *et al.* 2002; Dale and Moran 2006) modify enzyme structure in ways that increase permissiveness for use of alternate vitamin-like compounds. In such cases, predicted vitamin dependency may be ameliorated by increasingly flexible enzyme function.

The outcomes from this work also provide another perspective for considering the natural histories of symbiotic interaction. The ancient integration of β-proteobacterial endosymbionts (Bennett and Moran 2013) is corroborated by predictions of pervasive vitamin dependency, distinct from nonsymbiont members of this taxonomic group. Conversely, the similarity of vitamin dependency predictions between endosymbiont and nonsymbiont Tenericutes supports recent *Spiroplasma* introductions into honeybees, soldier beetles, and green-eyed horseflies (Moran *et al.* 2008). Portability of the analytical procedures presented here will create new opportunities to consider the metabolic framework of symbiosis and its implications across diverse systems, including plants (Bonfante and Anca 2009), cnidarians (Davy *et al.* 2012; Blackall *et al.* 2015), animals (Rader and Nyholm 2012), and protozoans (Nowack and Melkonian 2010). Adaptation of the workflow to analysis of other metabolic processes of interest, such as amino acid biosynthesis is also possible. In this age of “big data,” discussion of consensus analytical formats will facilitate broader molecular understanding of host-microbe interactions.

## Supplementary Material

Supplemental material is available online at www.g3journal.org/lookup/suppl/doi:10.1534/g3.117.042184/-/DC1.
