# Supplementary figures and images for: Predictive Genomic Analyses Inform the Basis for Vitamin Metabolism and Provisioning in Bacteria-Arthropod Endosymbioses
Source: G3 (Bethesda). 2017 Apr 28;7(6):1887–98. doi: 10.1534/g3.117.042184 (PMC5473766; doi:10.1534/g3.117.042184)

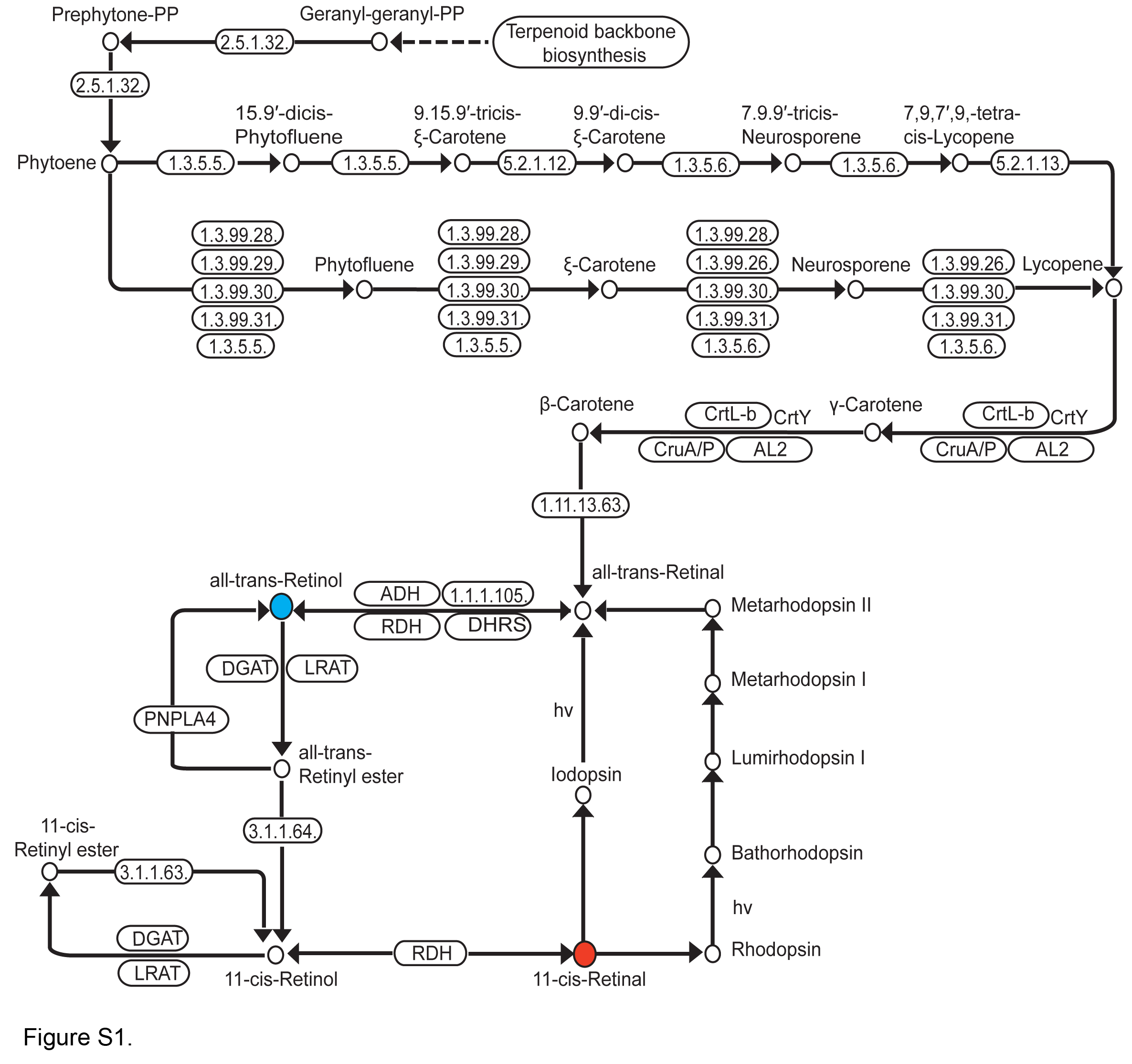

Supplement: Supplementary file 1 [file 1887FigureS1.tif]

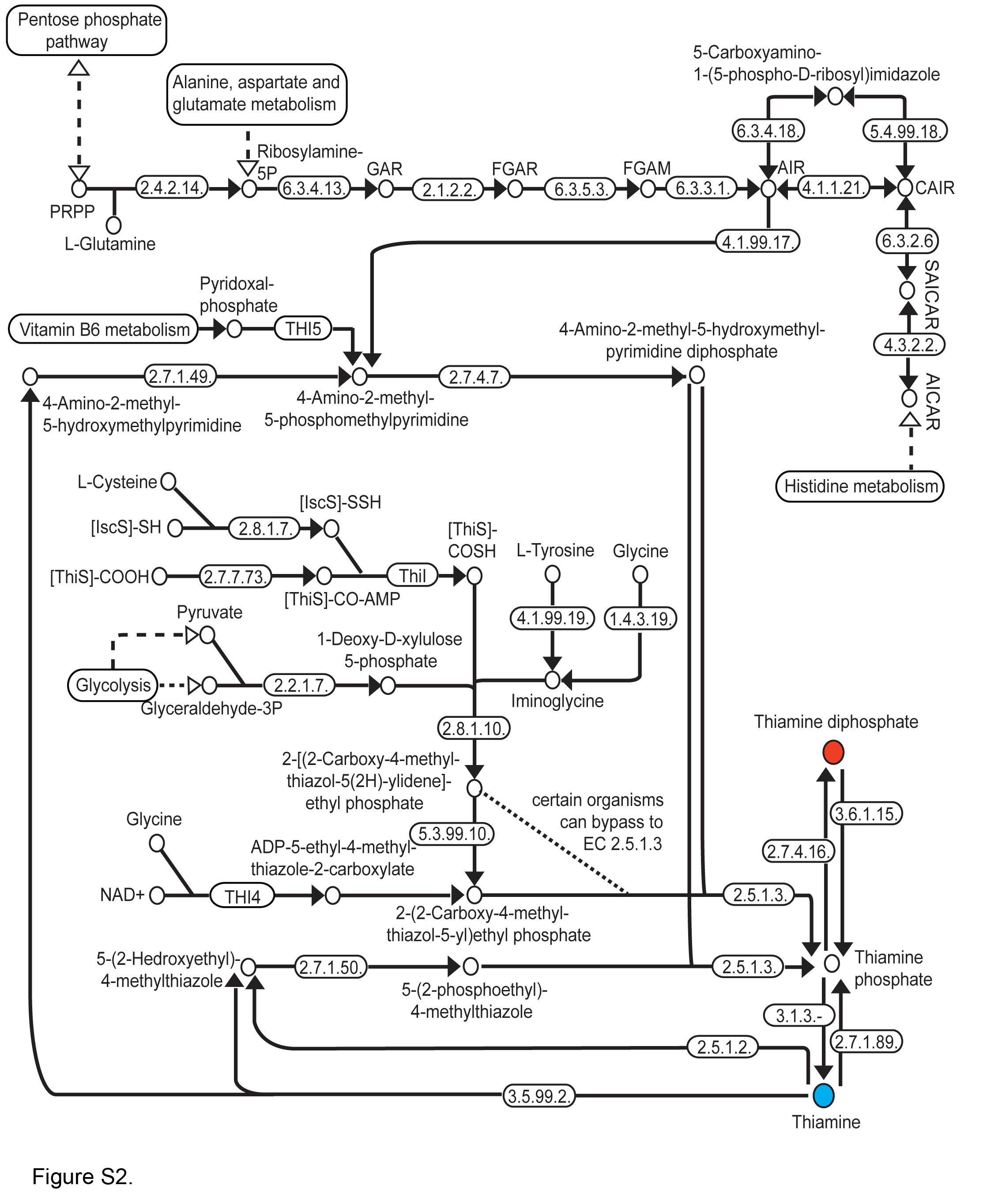

Supplement: Supplementary file 2 [file 1887FigureS2.tif]

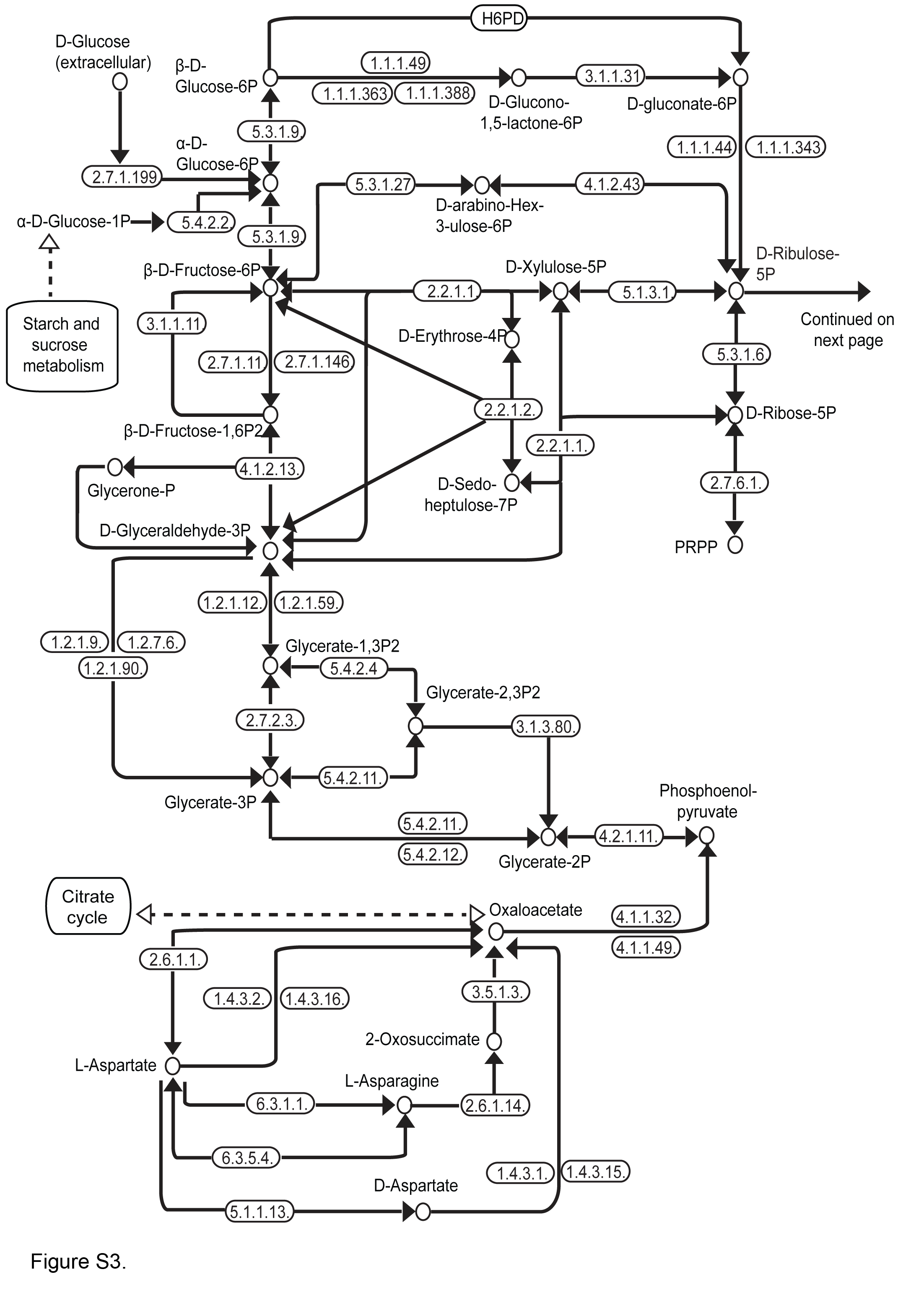

Supplement: Supplementary file 3 [file 1887FigureS3.tif]

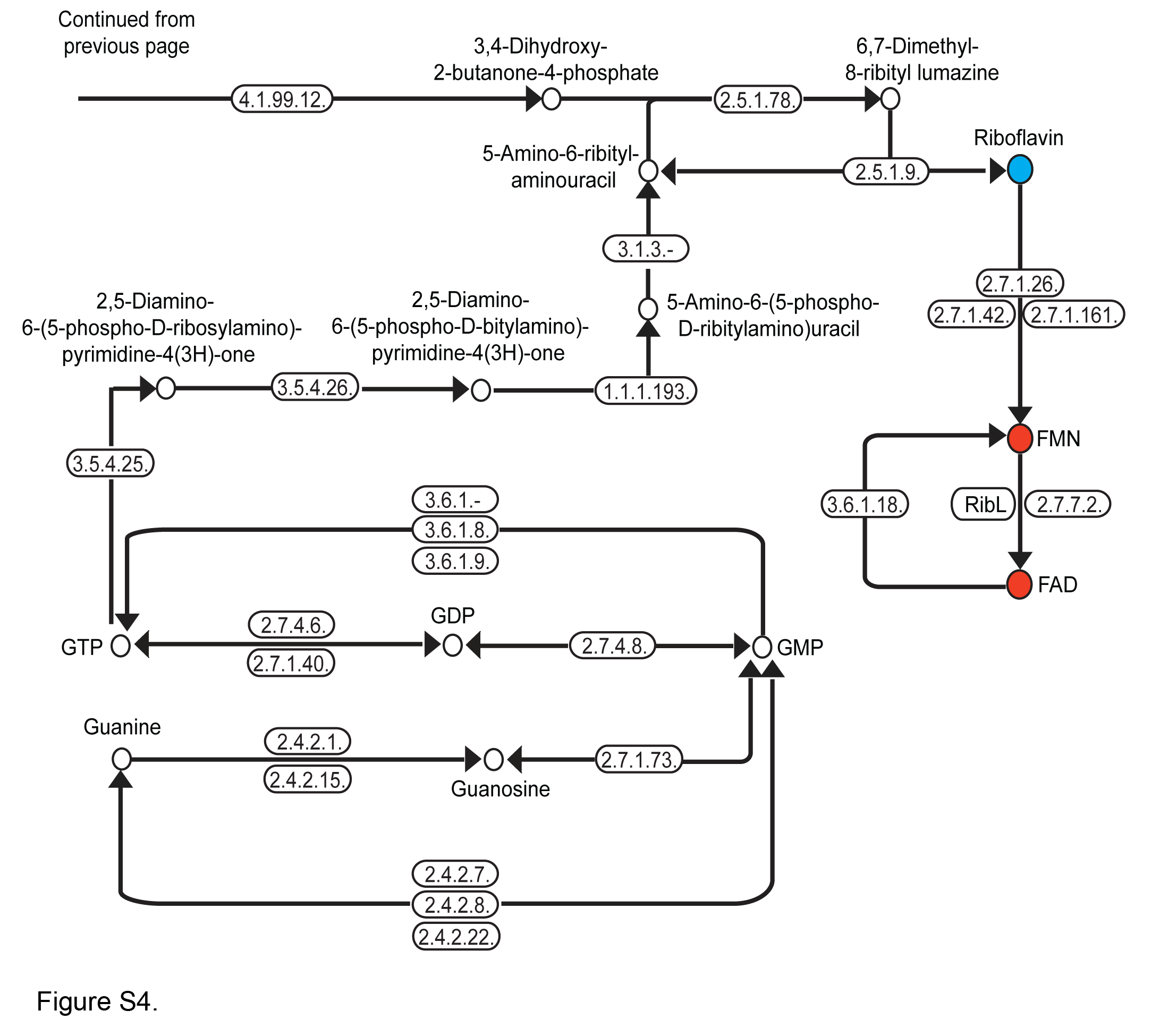

Supplement: Supplementary file 4 [file 1887FigureS4.tif]

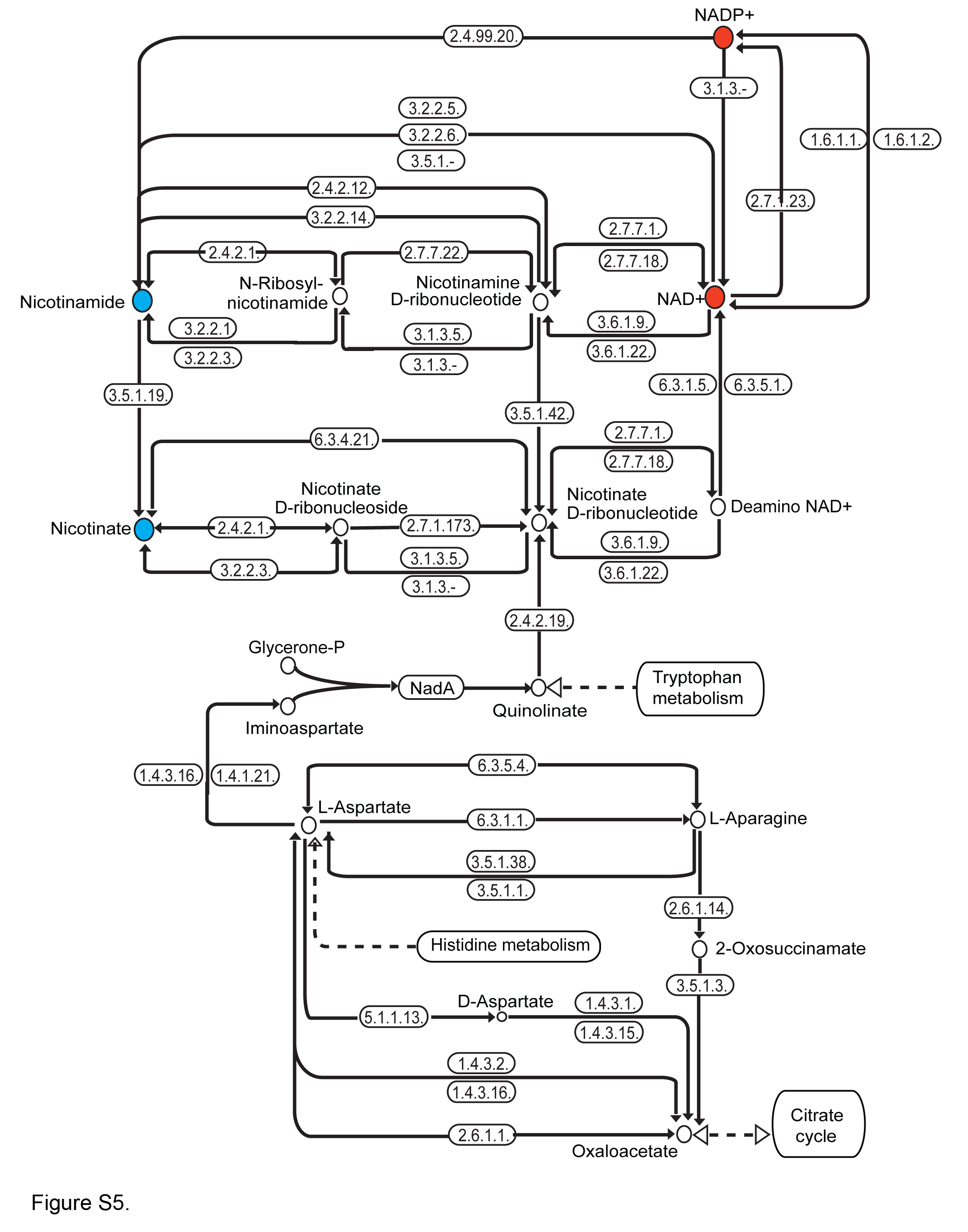

Supplement: Supplementary file 5 [file 1887FigureS5.tif]

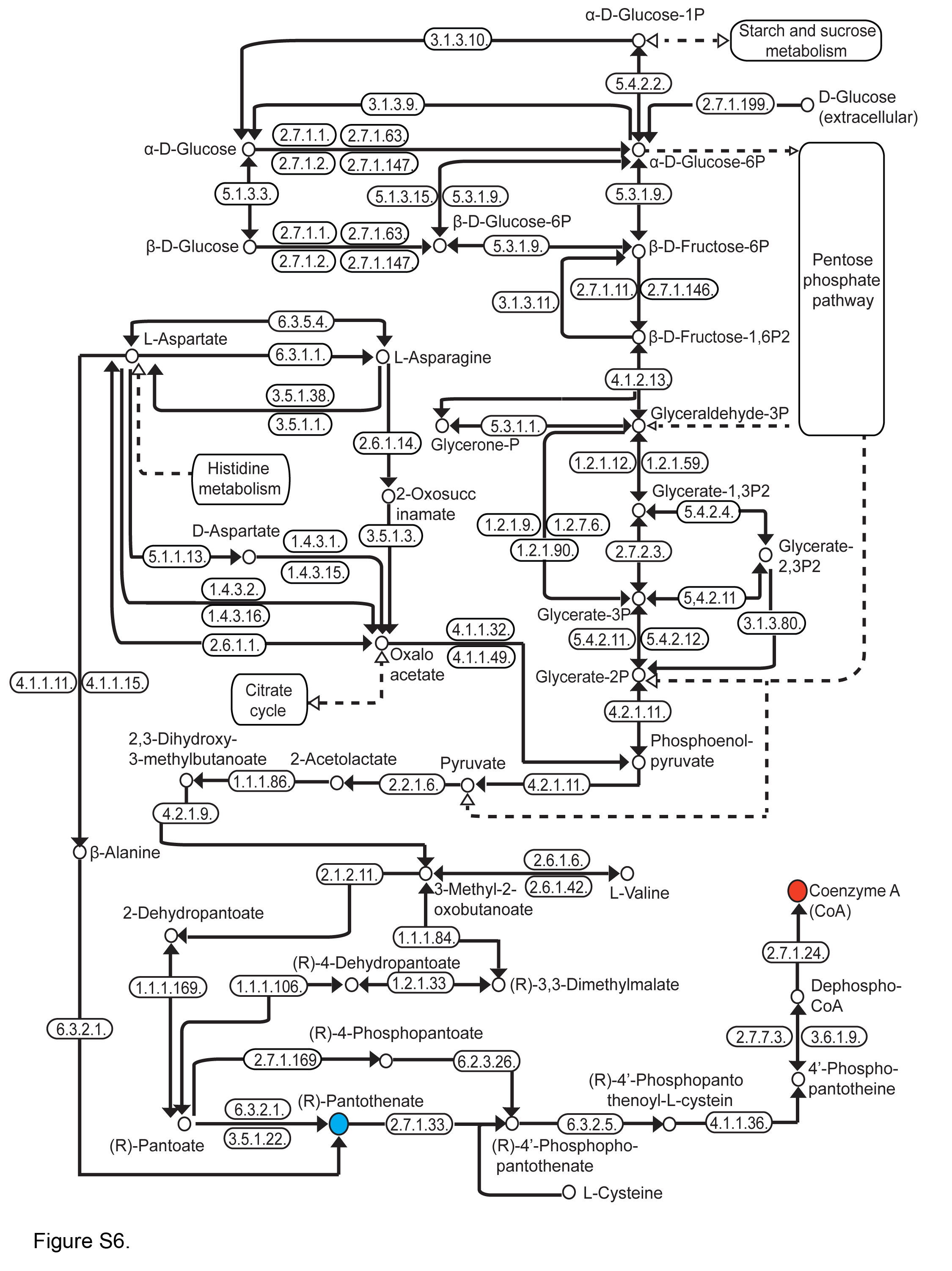

Supplement: Supplementary file 6 [file 1887FigureS6.tif]

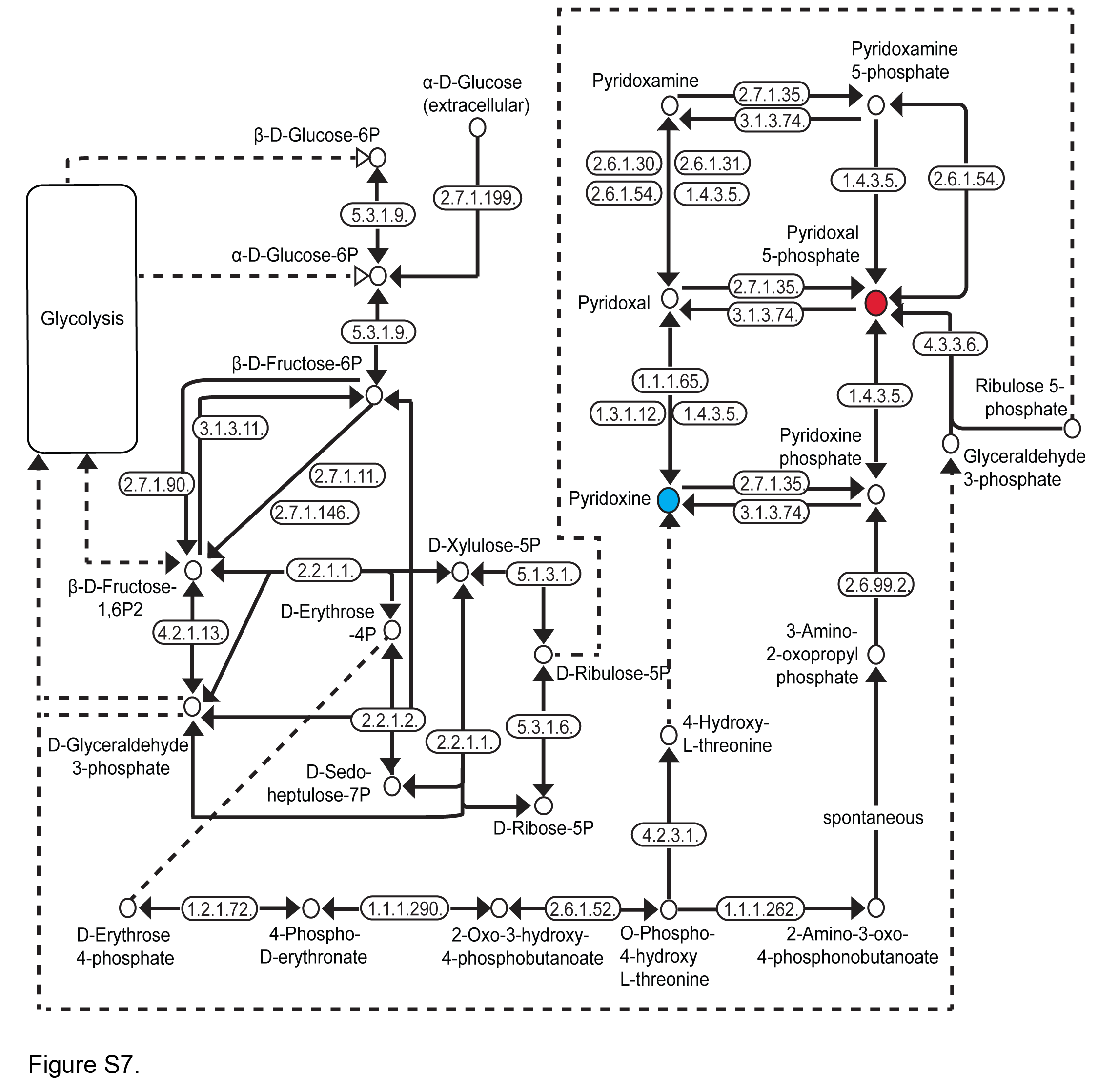

Supplement: Supplementary file 7 [file 1887FigureS7.tif]

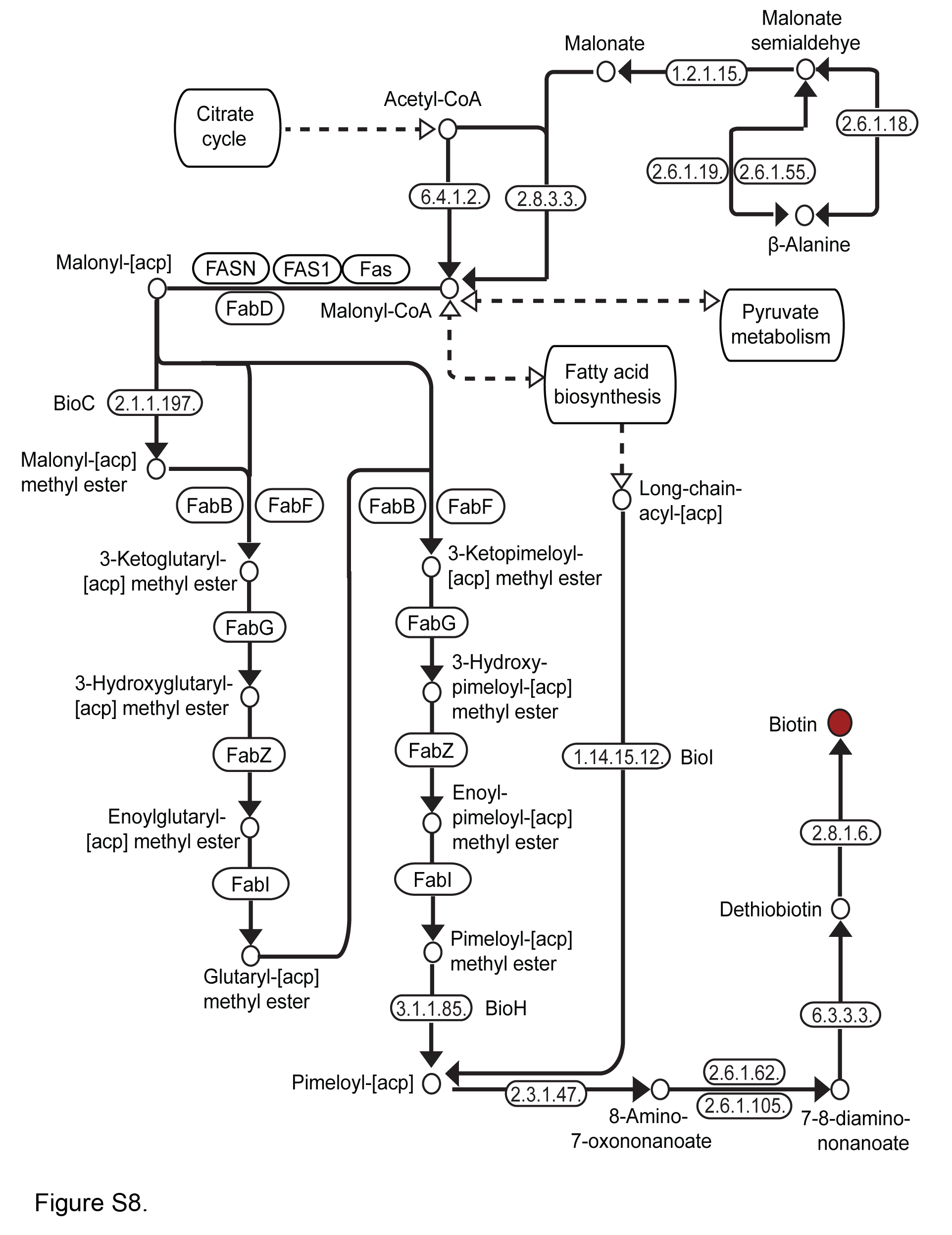

Supplement: Supplementary file 8 [file 1887FigureS8.tif]

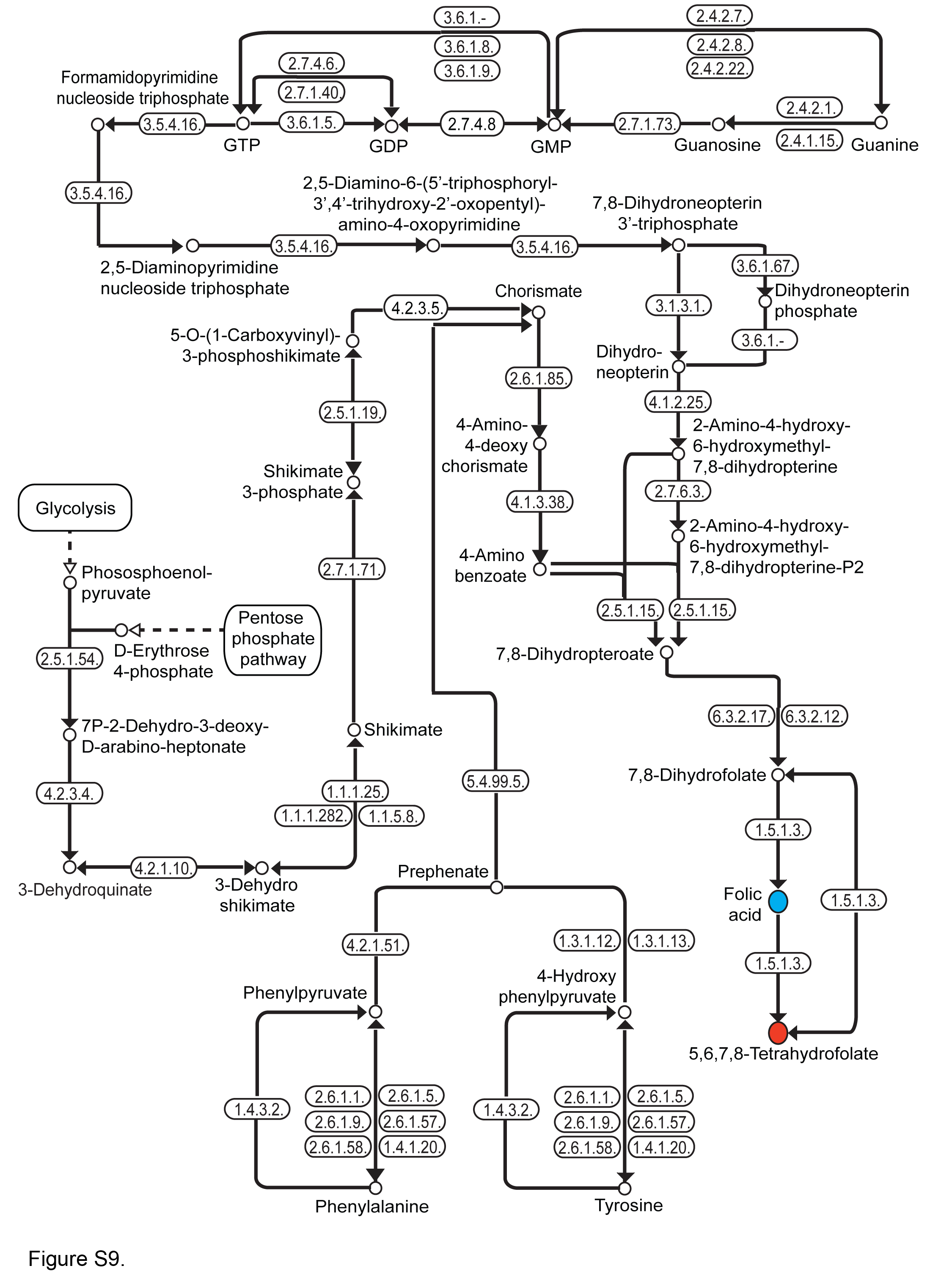

Supplement: Supplementary file 9 [file 1887FigureS9.tif]

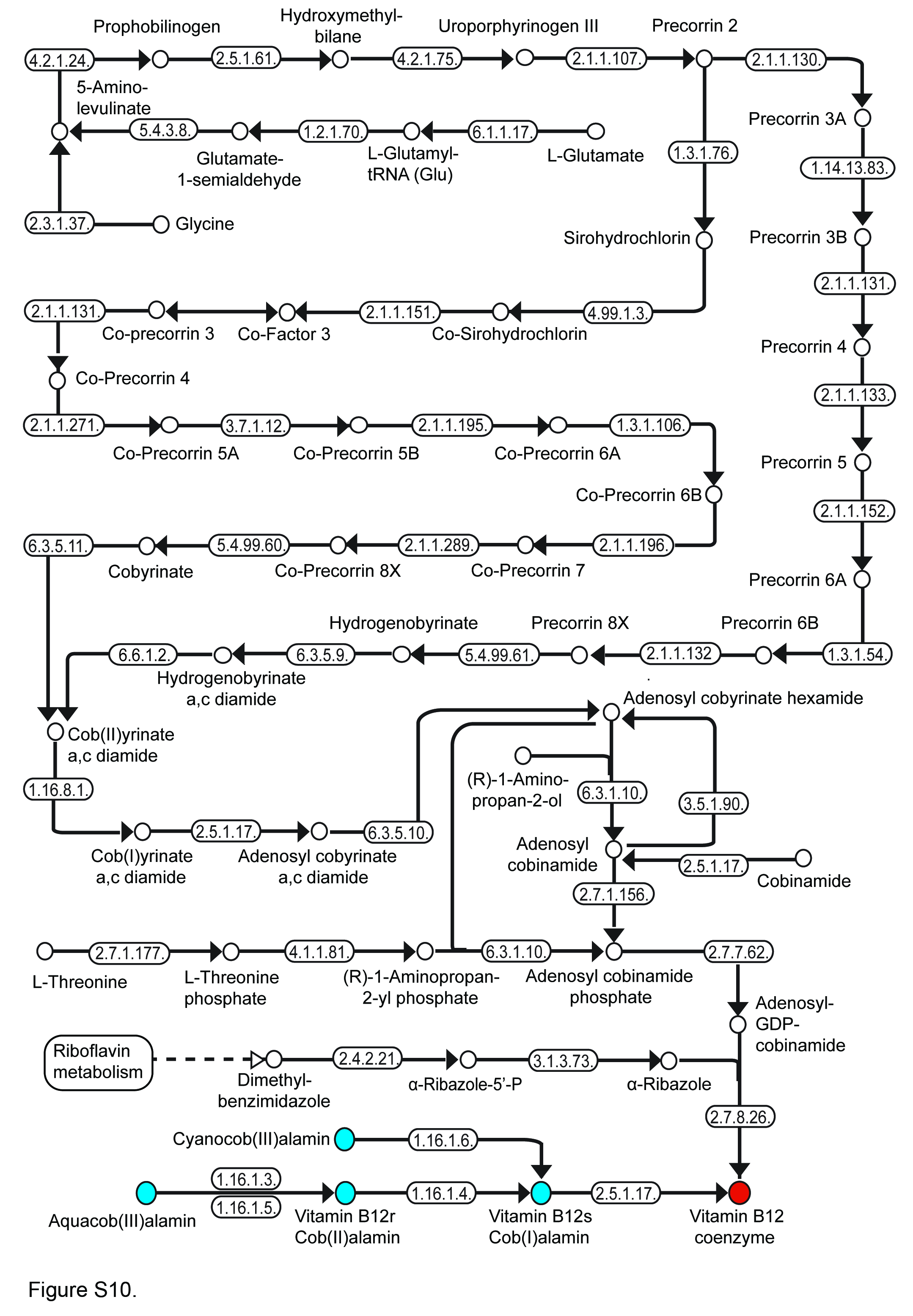

Supplement: Supplementary file 10 [file 1887FigureS10.tif]

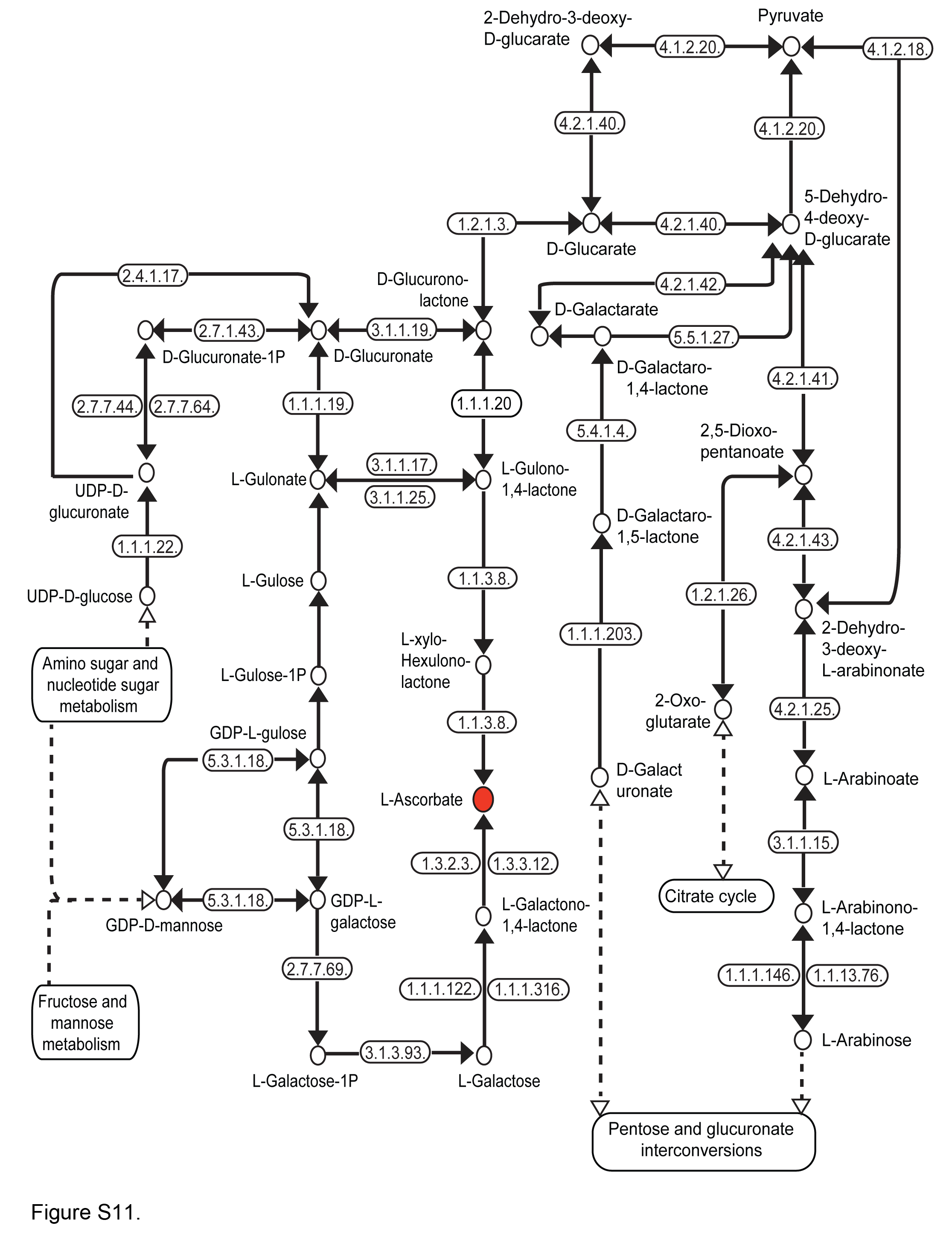

Supplement: Supplementary file 11 [file 1887FigureS11.tif]

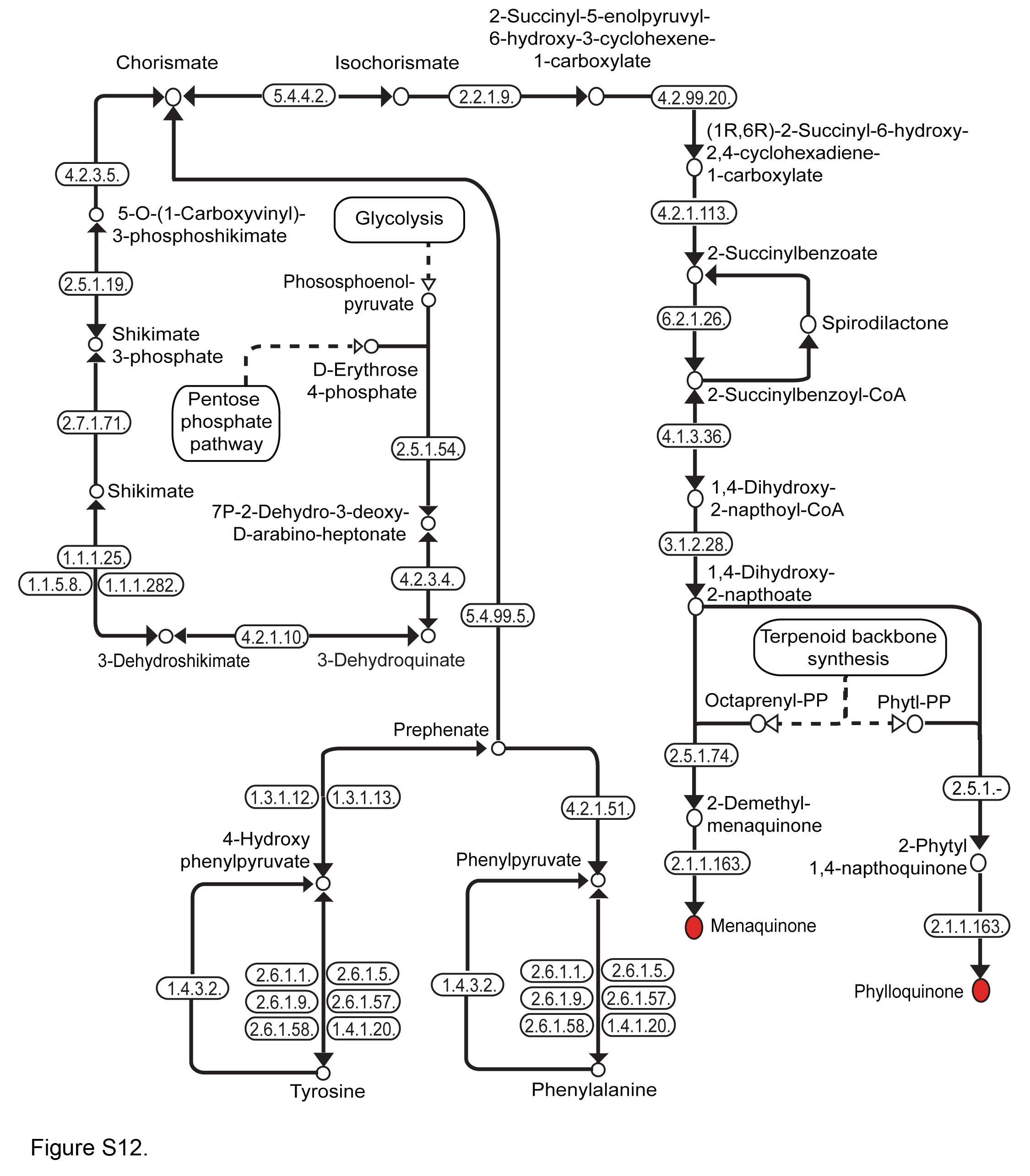

Supplement: Supplementary file 12 [file 1887FigureS12.tif]

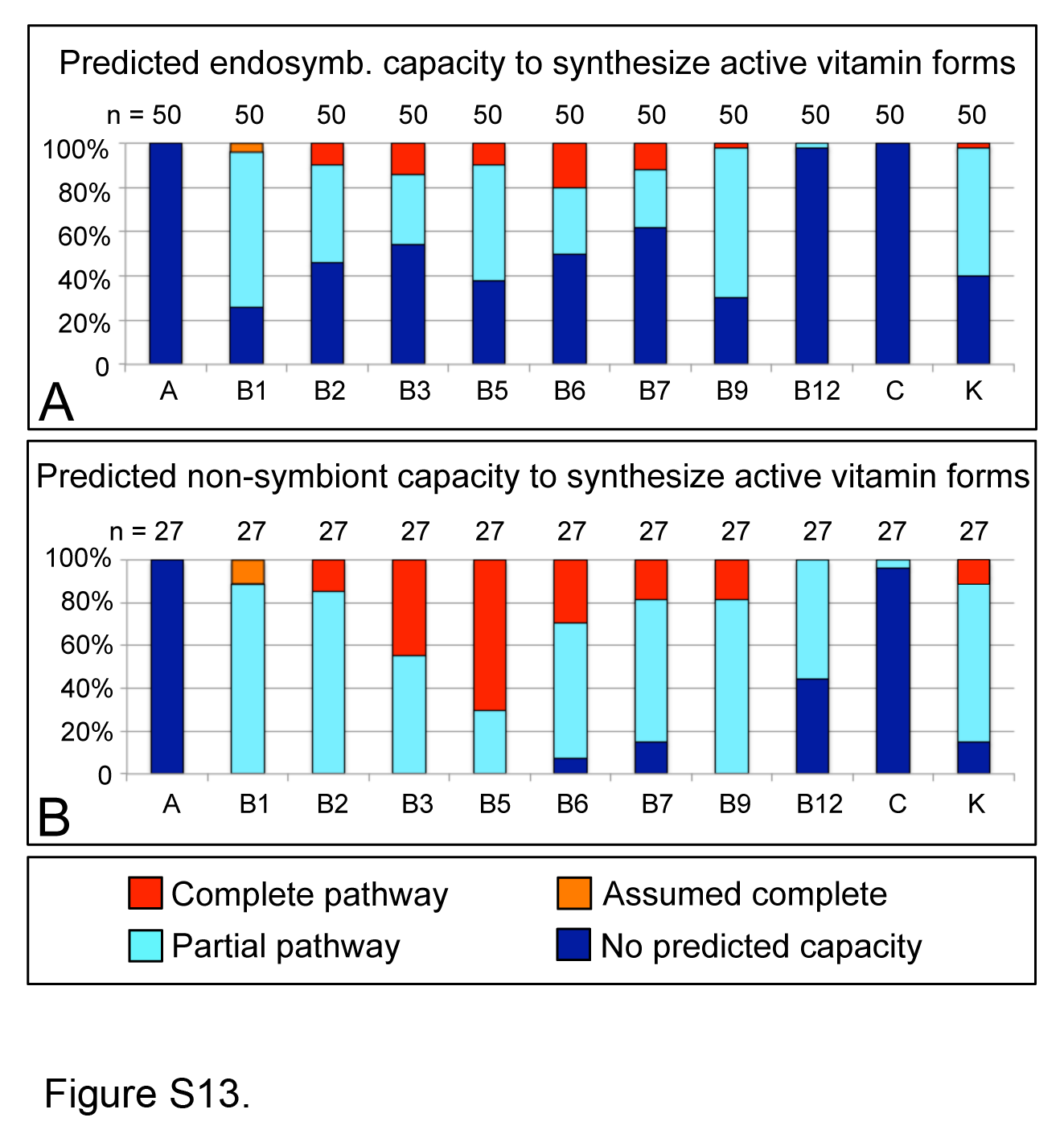

Supplement: Supplementary file 13 [file 1887FigureS13.tif]

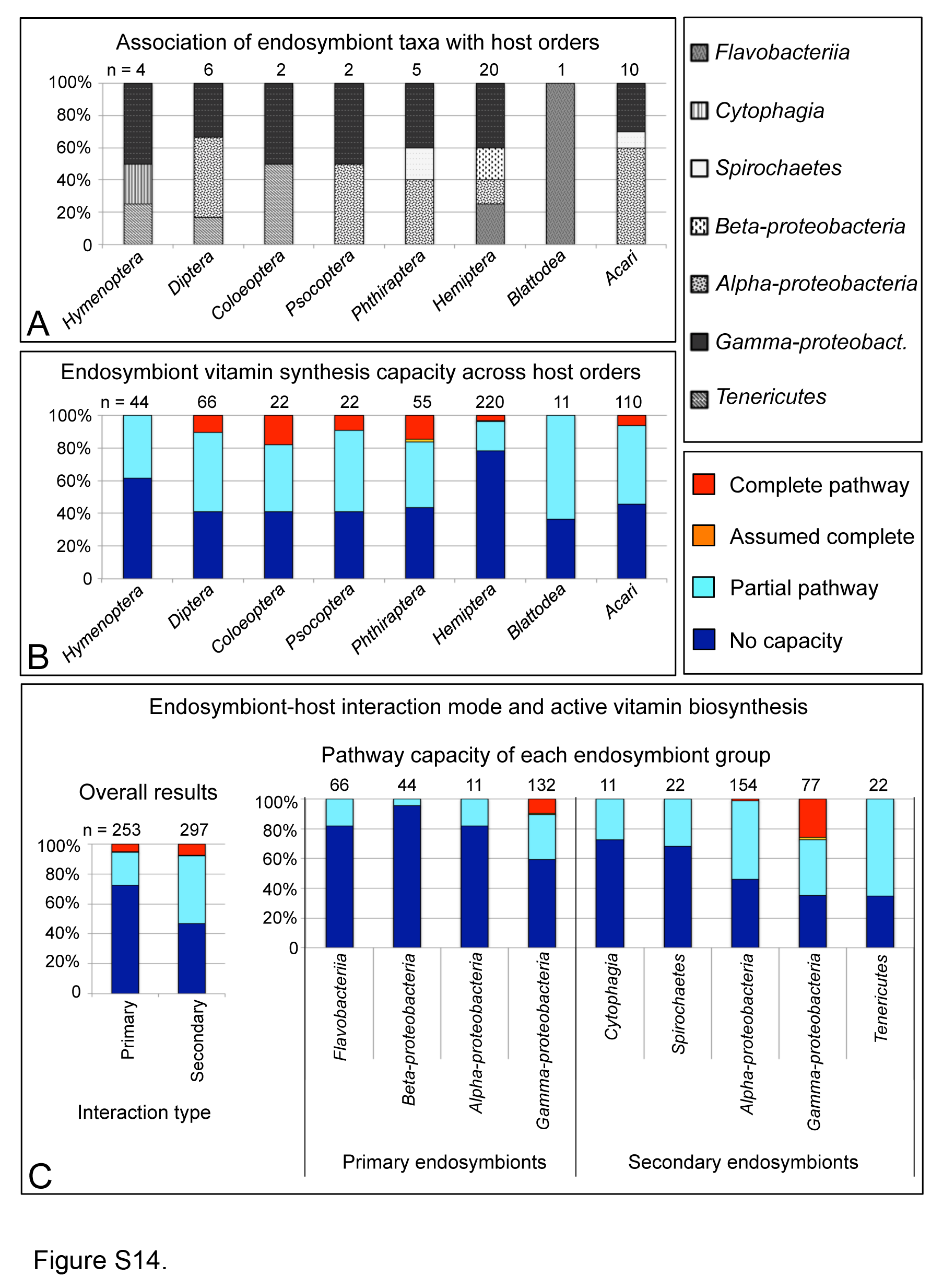

Supplement: Supplementary file 14 [file 1887FigureS14.tif]

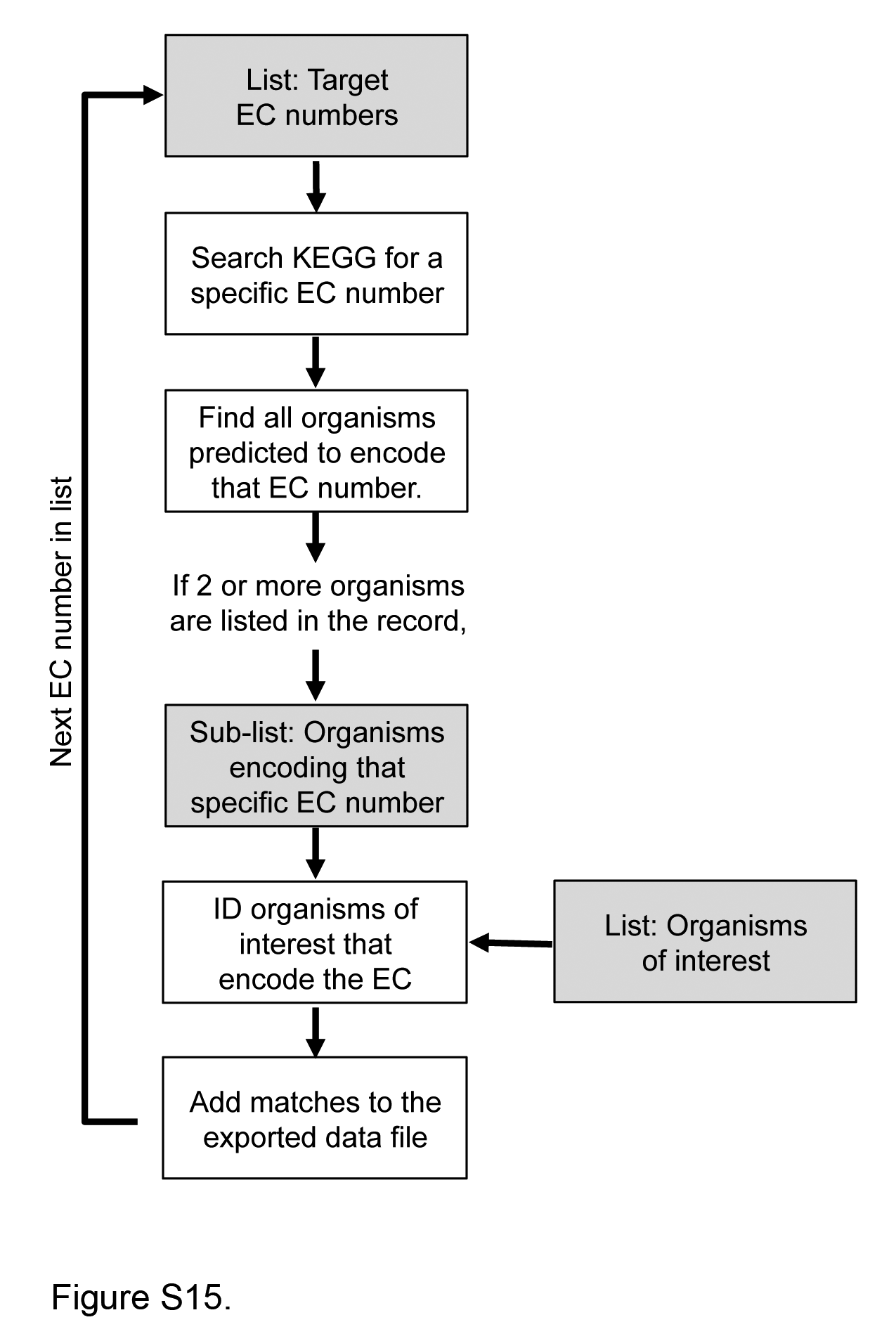

Supplement: Supplementary file 15 [file 1887FigureS15.tif]
