# Supplementary material for: Predictive Genomic Analyses Inform the Basis for Vitamin Metabolism and Provisioning in Bacteria-Arthropod Endosymbioses
Source: G3 (Bethesda). 2017 Apr 28;7(6):1887–98. doi: 10.1534/g3.117.042184 (PMC5473766; doi:10.1534/g3.117.042184)
Supplement: Supplementary file 16 [file 1887FileS1.docx]

**File S1: Supplementary References**

Abt, B., M. Goker, C. Scheuner, C. Han, M. Lu *et al.*, 2013 Genome sequence of the thermophilic fresh-water bacterium Spirochaeta caldaria type strain (H1(T)), reclassification of Spirochaeta caldaria, Spirochaeta stenostrepta, and Spirochaeta zuelzerae in the genus Treponema as Treponema caldaria comb. nov., Treponema stenostrepta comb. nov., and Treponema zuelzerae comb. nov., and emendation of the genus Treponema. *Stand Genomic Sci* 8 (1):88-105.

Akman, L., A. Yamashita, H. Watanabe, K. Oshima, T. Shiba *et al.*, 2002 Genome sequence of the endocellular obligate symbiont of tsetse flies, Wigglesworthia glossinidia. *Nat Genet* 32 (3):402-407.

Alsmark, C.M., A.C. Frank, E.O. Karlberg, B.A. Legault, D.H. Ardell *et al.*, 2004 The louse-borne human pathogen Bartonella quintana is a genomic derivative of the zoonotic agent Bartonella henselae. *Proc Natl Acad Sci U S A* 101 (26):9716-9721.

Barbier, P., A. Houel, V. Loux, J. Poulain, J.F. Bernardet *et al.*, 2012 Complete genome sequence of Flavobacterium indicum GPSTA100-9T, isolated from warm spring water. *J Bacteriol* 194 (11):3024-3025.

Bechah, Y., K. El Karkouri, O. Mediannikov, Q. Leroy, N. Pelletier *et al.*, 2010 Genomic, proteomic, and transcriptomic analysis of virulent and avirulent Rickettsia prowazekii reveals its adaptive mutation capabilities. *Genome Res* 20 (5):655-663.

Beller, H.R., P.S. Chain, T.E. Letain, A. Chakicherla, F.W. Larimer *et al.*, 2006 The genome sequence of the obligately chemolithoautotrophic, facultatively anaerobic bacterium Thiobacillus denitrificans. *J Bacteriol* 188 (4):1473-1488.

Ben Hania, W., M. Joseph, P. Schumann, B. Bunk, A. Fiebig *et al.*, 2015 Complete genome sequence and description of Salinispira pacifica gen. nov., sp. nov., a novel spirochaete isolated form a hypersaline microbial mat. *Stand Genomic Sci* 10:7.

Bennett, G.M., and N.A. Moran, 2013 Small, smaller, smallest: the origins and evolution of ancient dual symbioses in a Phloem-feeding insect. *Genome Biol Evol* 5 (9):1675-1688.

Blattner, F.R., G. Plunkett, 3rd, C.A. Bloch, N.T. Perna, V. Burland *et al.*, 1997 The complete genome sequence of Escherichia coli K-12. *Science* 277 (5331):1453-1462.

Brazilian National Genome Project, C., 2003 The complete genome sequence of Chromobacterium violaceum reveals remarkable and exploitable bacterial adaptability. *Proc Natl Acad Sci U S A* 100 (20):11660-11665.

Chain, P., J. Lamerdin, F. Larimer, W. Regala, V. Lao *et al.*, 2003 Complete genome sequence of the ammonia-oxidizing bacterium and obligate chemolithoautotroph Nitrosomonas europaea. *J Bacteriol* 185 (9):2759-2773.

Dai, J., W. Dai, C. Qiu, Z. Yang, Y. Zhang *et al.*, 2015 Unraveling adaptation of Pontibacter korlensis to radiation and infertility in desert through complete genome and comparative transcriptomic analysis. *Sci Rep* 5:10929.

Degnan, P.H., Y. Yu, N. Sisneros, R.A. Wing, and N.A. Moran, 2009 Hamiltonella defensa, genome evolution of protective bacterial endosymbiont from pathogenic ancestors. *Proc Natl Acad Sci U S A* 106 (22):9063-9068.

Duan, Y., L. Zhou, D.G. Hall, W. Li, H. Doddapaneni *et al.*, 2009 Complete genome sequence of citrus huanglongbing bacterium, 'Candidatus Liberibacter asiaticus' obtained through metagenomics. *Mol Plant Microbe Interact* 22 (8):1011-1020.

Dunning Hotopp, J.C., M. Lin, R. Madupu, J. Crabtree, S.V. Angiuoli *et al.*, 2006 Comparative genomics of emerging human ehrlichiosis agents. *Plos Genetics* 2 (2):e21.

Eppinger, M., P.L. Worsham, M.P. Nikolich, D.R. Riley, Y. Sebastian *et al.*, 2010 Genome sequence of the deep-rooted Yersinia pestis strain Angola reveals new insights into the evolution and pangenome of the plague bacterium. *J Bacteriol* 192 (6):1685-1699.

Felsheim, R.F., S.L. Johnson, T.J. Kurtti, and U.G. Munderloh, 2015 Direct submission to NCBI: Draft genome sequence of Rickettsia monacensis strain IrR/Munich.

Fraser, C.M., S. Casjens, W.M. Huang, G.G. Sutton, R. Clayton *et al.*, 1997 Genomic sequence of a Lyme disease spirochaete, Borrelia burgdorferi. *Nature* 390 (6660):580-586.

Ghosh, W., A. George, A. Agarwal, P. Raj, M. Alam *et al.*, 2011 Whole-genome shotgun sequencing of the sulfur-oxidizing chemoautotroph Tetrathiobacter kashmirensis. *J Bacteriol* 193 (19):5553-5554.

Gil, R., F.J. Silva, E. Zientz, F. Delmotte, F. Gonzalez-Candelas *et al.*, 2003 The genome sequence of Blochmannia floridanus: comparative analysis of reduced genomes. *Proc Natl Acad Sci U S A* 100 (16):9388-9393.

Gottlieb, Y., I. Lalzar, and L. Klasson, 2015 Distinctive Genome Reduction Rates Revealed by Genomic Analyses of Two Coxiella-Like Endosymbionts in Ticks. *Genome Biol Evol* 7 (6):1779-1796.

Heidelberg, J.F., I.T. Paulsen, K.E. Nelson, E.J. Gaidos, W.C. Nelson *et al.*, 2002 Genome sequence of the dissimilatory metal ion-reducing bacterium Shewanella oneidensis. *Nat Biotechnol* 20 (11):1118-1123.

Huo, Y.Y., H. Cheng, X.F. Han, X.W. Jiang, C. Sun *et al.*, 2012 Complete genome sequence of Pelagibacterium halotolerans B2(T). *J Bacteriol* 194 (1):197-198.

Johnson, S.L., A.C. Munk, S. Han, D.C. Bruce, and G.A. Dasch, 2016 Direct submission to NCBI: Candidatus Rickettsia amblyommii str. GAT-30V, complete genome.

Kirkness, E.F., B.J. Haas, W. Sun, H.R. Braig, M.A. Perotti *et al.*, 2010 Genome sequences of the human body louse and its primary endosymbiont provide insights into the permanent parasitic lifestyle. *Proc Natl Acad Sci U S A* 107 (27):12168-12173.

Klasson, L., T. Walker, M. Sebaihia, M.J. Sanders, M.A. Quail *et al.*, 2008 Genome evolution of *Wolbachia* strain *w*Pip from the *Culex pipiens* group. *Molecular biology and evolution* 25 (9):1877-1887.

Klasson, L., J. Westberg, P. Sapountzis, K. Naslund, Y. Lutnaes *et al.*, 2009 The mosaic genome structure of the Wolbachia wRi strain infecting Drosophila simulans. *Proc Natl Acad Sci U S A* 106 (14):5725-5730.

Koga, R., and N.A. Moran, 2014 Swapping symbionts in spittlebugs: evolutionary replacement of a reduced genome symbiont. *ISME J* 8 (6):1237-1246.

Ku, C., W.S. Lo, L.L. Chen, and C.H. Kuo, 2014 Complete Genome Sequence of Spiroplasma apis B31T (ATCC 33834), a Bacterium Associated with May Disease of Honeybees (Apis mellifera). *Genome Announc* 2 (1).

Lazarev, V.N., S.A. Levitskii, Y.I. Basovskii, M.M. Chukin, T.A. Akopian *et al.*, 2011 Complete genome and proteome of Acholeplasma laidlawii. *J Bacteriol* 193 (18):4943-4953.

Lescot, M., S. Audic, C. Robert, T.T. Nguyen, G. Blanc *et al.*, 2008 The genome of Borrelia recurrentis, the agent of deadly louse-borne relapsing fever, is a degraded subset of tick-borne Borrelia duttonii. *Plos Genetics* 4 (9):e1000185.

Lo, W.S., Y.C. Lai, Y.W. Lien, T.H. Wang, and C.H. Kuo, 2015a Complete Genome Sequence of Spiroplasma litorale TN-1T (DSM 21781), a Bacterium Isolated from a Green-Eyed Horsefly (Tabanus nigrovittatus). *Genome Announc* 3 (5).

Lo, W.S., P.Y. Liu, and C.H. Kuo, 2015b Complete Genome Sequence of Spiroplasma cantharicola CC-1T (DSM 21588), a Bacterium Isolated from Soldier Beetle (Cantharis carolinus). *Genome Announc* 3 (5).

Lopez-Madrigal, S., A. Latorre, M. Porcar, A. Moya, and R. Gil, 2011 Complete genome sequence of "Candidatus Tremblaya princeps" strain PCVAL, an intriguing translational machine below the living-cell status. *J Bacteriol* 193 (19):5587-5588.

Lucas, S., A. Copeland, A. Lapidus, J.-F. Cheng, L. Goodwin *et al.*, 2015 Direct submission to NCBI: Complete sequence of Sphaerochaeta globosa Buddy.

Lucas, S., A. Copeland, A. Lapidus, T. Glavina del Rio, E. Dalin *et al.*, 2017 Direct submission to NCBI: The complete genome of Flexibacter litoralis DSM 6794.

Marks, M.E., C.M. Castro-Rojas, C. Teiling, L. Du, V. Kapatral *et al.*, 2010 The genetic basis of laboratory adaptation in Caulobacter crescentus. *J Bacteriol* 192 (14):3678-3688.

Mavromatis, K., M. Yasawong, O. Chertkov, A. Lapidus, S. Lucas *et al.*, 2010 Complete genome sequence of Spirochaeta smaragdinae type strain (SEBR 4228). *Stand Genomic Sci* 3 (2):136-144.

McCutcheon, J.P., B.R. McDonald, and N.A. Moran, 2009a Convergent evolution of metabolic roles in bacterial co-symbionts of insects. *Proc Natl Acad Sci U S A* 106 (36):15394-15399.

McCutcheon, J.P., B.R. McDonald, and N.A. Moran, 2009b Origin of an alternative genetic code in the extremely small and GC-rich genome of a bacterial symbiont. *Plos Genetics* 5 (7):e1000565.

McCutcheon, J.P., and N.A. Moran, 2007 Parallel genomic evolution and metabolic interdependence in an ancient symbiosis. *Proc Natl Acad Sci U S A* 104 (49):19392-19397.

McCutcheon, J.P., and N.A. Moran, 2010 Functional convergence in reduced genomes of bacterial symbionts spanning 200 My of evolution. *Genome Biol Evol* 2:708-718.

McCutcheon, J.P., and C.D. von Dohlen, 2011 An interdependent metabolic patchwork in the nested symbiosis of mealybugs. *Current Biology* 21 (16):1366-1372.

Modise, T., C. Ryder, S.P. Mane, A.B. Bandara, R.V. Jensen *et al.*, 2017 Direct submission to NCBI: Francisella tularensis TI0902, complete genome.

Muraguchi, Y., K. Kushimoto, Y. Ohtsubo, T. Suzuki, H. Dohra *et al.*, 2016 Complete Genome Sequence of Algoriphagus sp. Strain M8-2, Isolated from a Brackish Lake. *Genome Announc* 4 (3).

Nakabachi, A., R. Ueoka, K. Oshima, R. Teta, A. Mangoni *et al.*, 2013 Defensive bacteriome symbiont with a drastically reduced genome. *Current Biology* 23 (15):1478-1484.

Nakabachi, A., A. Yamashita, H. Toh, H. Ishikawa, H.E. Dunbar *et al.*, 2006 The 160-kilobase genome of the bacterial endosymbiont Carsonella. *Science* 314 (5797):267.

Nakayama, K., A. Yamashita, K. Kurokawa, T. Morimoto, M. Ogawa *et al.*, 2008 The Whole-genome sequencing of the obligate intracellular bacterium Orientia tsutsugamushi revealed massive gene amplification during reductive genome evolution. *DNA Res* 15 (4):185-199.

Nikoh, N., T. Hosokawa, M. Moriyama, K. Oshima, M. Hattori *et al.*, 2014 Evolutionary origin of insect–*Wolbachia* nutritional mutualism. *Proceedings of the National Academy of Sciences* 111 (28):10257-10262.

Novakova, E., V. Hypsa, P. Nguyen, F. Husnik, and A.C. Darby, 2016 Genome sequence of Candidatus Arsenophonus lipopteni, the exclusive symbiont of a blood sucking fly Lipoptena cervi (Diptera: Hippoboscidae). *Stand Genomic Sci* 11:72.

Oakeson, K.F., R. Gil, A.L. Clayton, D.M. Dunn, A.C. von Niederhausern *et al.*, 2014 Genome degeneration and adaptation in a nascent stage of symbiosis. *Genome Biol Evol* 6 (1):76-93.

Ogata, H., P. Renesto, S. Audic, C. Robert, G. Blanc *et al.*, 2005 The genome sequence of Rickettsia felis identifies the first putative conjugative plasmid in an obligate intracellular parasite. *PLoS Biol* 3 (8):e248.

Oh, H.M., S.J. Giovannoni, S. Ferriera, J. Johnson, and J.C. Cho, 2009 Complete genome sequence of Erythrobacter litoralis HTCC2594. *J Bacteriol* 191 (7):2419-2420.

Penz, T., S. Schmitz-Esser, S.E. Kelly, B.N. Cass, A. Muller *et al.*, 2012 Comparative genomics suggests an independent origin of cytoplasmic incompatibility in Cardinium hertigii. *Plos Genetics* 8 (10):e1003012.

Perez-Brocal, V., R. Gil, S. Ramos, A. Lamelas, M. Postigo *et al.*, 2006 A small microbial genome: the end of a long symbiotic relationship? *Science* 314 (5797):312-313.

Qin, Q.L., X.Y. Zhang, X.M. Wang, G.M. Liu, X.L. Chen *et al.*, 2010 The complete genome of Zunongwangia profunda SM-A87 reveals its adaptation to the deep-sea environment and ecological role in sedimentary organic nitrogen degradation. *Bmc Genomics* 11:247.

Riedel, T., B. Held, M. Nolan, S. Lucas, A. Lapidus *et al.*, 2012 Genome sequence of the orange-pigmented seawater bacterium Owenweeksia hongkongensis type strain (UST20020801(T)). *Stand Genomic Sci* 7 (1):120-130.

Sabree, Z.L., C.Y. Huang, A. Okusu, N.A. Moran, and B.B. Normark, 2013 The nutrient supplying capabilities of Uzinura, an endosymbiont of armoured scale insects. *Environ Microbiol* 15 (7):1988-1999.

Sabree, Z.L., S. Kambhampati, and N.A. Moran, 2009 Nitrogen recycling and nutritional provisioning by Blattabacterium, the cockroach endosymbiont. *Proc Natl Acad Sci U S A* 106 (46):19521-19526.

Santos-Garcia, D., P.A. Farnier, F. Beitia, E. Zchori-Fein, F. Vavre *et al.*, 2012 Complete genome sequence of "Candidatus Portiera aleyrodidarum" BT-QVLC, an obligate symbiont that supplies amino acids and carotenoids to Bemisia tabaci. *J Bacteriol* 194 (23):6654-6655.

Schwibbert, K., A. Marin-Sanguino, I. Bagyan, G. Heidrich, G. Lentzen *et al.*, 2011 A blueprint of ectoine metabolism from the genome of the industrial producer Halomonas elongata DSM 2581 T. *Environ Microbiol* 13 (8):1973-1994.

Shigenobu, S., H. Watanabe, M. Hattori, Y. Sakaki, and H. Ishikawa, 2000 Genome sequence of the endocellular bacterial symbiont of aphids Buchnera sp. APS. *Nature* 407 (6800):81-86.

Skennerton, C.T., M.F. Haroon, A. Briegel, J. Shi, G.J. Jensen *et al.*, 2016 Phylogenomic analysis of Candidatus 'Izimaplasma' species: free-living representatives from a Tenericutes clade found in methane seeps. *ISME J* 10 (11):2679-2692.

Sloan, D.B., and N.A. Moran, 2012 Endosymbiotic bacteria as a source of carotenoids in whiteflies. *Biol Lett* 8 (6):986-989.

Smith, T.A., T. Driscoll, J.J. Gillespie, and R. Raghavan, 2015 A Coxiella-like endosymbiont is a potential vitamin source for the Lone Star tick. *Genome Biol Evol* 7 (3):831-838.

Snopkova, K., K. Sedlar, J. Bosak, E. Chaloupkova, I. Provaznik *et al.*, 2015 Complete Genome Sequence of Pragia fontium 24613, an Environmental Bacterium from the Family Enterobacteriaceae. *Genome Announc* 3 (4).

Swarnkar, M.K., A. Sharma, K. Kaushal, R. Soni, S. Rana *et al.*, 2016 Direct submission to NCBI: Complete Genome Sequence of Halotalea alkalilenta IHB B 13600.

Toh, H., B.L. Weiss, S.A. Perkin, A. Yamashita, K. Oshima *et al.*, 2006 Massive genome erosion and functional adaptations provide insights into the symbiotic lifestyle of Sodalis glossinidius in the tsetse host. *Genome Res* 16 (2):149-156.

Volland, S., M. Rachinger, A. Strittmatter, R. Daniel, G. Gottschalk *et al.*, 2011 Complete genome sequences of the chemolithoautotrophic Oligotropha carboxidovorans strains OM4 and OM5. *J Bacteriol* 193 (18):5043.

Williams, L.E., and J.J. Wernegreen, 2015 Genome evolution in an ancient bacteria-ant symbiosis: parallel gene loss among Blochmannia spanning the origin of the ant tribe Camponotini. *PeerJ* 3:e881.

Woyke, T., O. Chertkov, A. Lapidus, M. Nolan, S. Lucas *et al.*, 2011 Complete genome sequence of the gliding freshwater bacterium Fluviicola taffensis type strain (RW262). *Stand Genomic Sci* 5 (1):21-29.

Wu, D., S.C. Daugherty, S.E. Van Aken, G.H. Pai, K.L. Watkins *et al.*, 2006 Metabolic complementarity and genomics of the dual bacterial symbiosis of sharpshooters. *PLoS Biol* 4 (6):e188.

Wu, M., L.V. Sun, J. Vamathevan, M. Riegler, R. Deboy *et al.*, 2004 Phylogenomics of the reproductive parasite *Wolbachia pipientis w*Mel: a streamlined genome overrun by mobile genetic elements. *PLoS Biol* 2 (3):E69.

Xie, G., D.C. Bruce, J.F. Challacombe, O. Chertkov, J.C. Detter *et al.*, 2007 Genome sequence of the cellulolytic gliding bacterium Cytophaga hutchinsonii. *Appl Environ Microbiol* 73 (11):3536-3546.
