# Supplementary material for: Predictive Genomic Analyses Inform the Basis for Vitamin Metabolism and Provisioning in Bacteria-Arthropod Endosymbioses
Source: G3 (Bethesda). 2017 Apr 28;7(6):1887–98. doi: 10.1534/g3.117.042184 (PMC5473766; doi:10.1534/g3.117.042184)
Supplement: Supplementary file 23 [file 1887FileS8.docx]

**SUPPLEMENTARY FIGURE LEGENDS**

**Figure S1.** Pathway activity that supports vitamin A synthesis. For this and all subsequent pathway maps, the EC numbers are enclosed by ovals and the products are indicated as circles with names to one side. Blue circle: conventional vitamin product. Red circle: active vitamin form. Converging lines indicate that both input products are necessary for the next step of synthesis. Divergent lines indicate production of multiple products. Bidirectional arrows indicate reversible reactions. This content represents adapted, customized forms of publicly available KEGG pathway maps, referenced in Table S3.

**Figure S2.** Pathway activity supporting vitamin B1 (Thiamine) biosynthesis. Blue circle: conventional vitamin product. Red circle: active vitamin form. This content represents adapted, customized forms of publicly available KEGG pathway maps, referenced in Table S3.

**Figure S3.** Part 1: Pathway activity supporting vitamin B2 (Riboflavin) biosynthesis. This content represents adapted, customized forms of publicly available KEGG pathway maps, referenced in Table S3.

**Figure S4.** Part 2: Pathway activity supporting vitamin B2 (Riboflavin) biosynthesis.

Blue circle: conventional vitamin product. Red circles: active vitamin forms. This content represents adapted, customized forms of publicly available KEGG pathway maps, referenced in Table S3.

**Figure S5.** Pathway activity supporting vitamin B3 (Nicotinate) biosynthesis. Blue circle: conventional vitamin products. Red circle: active vitamin forms. This content represents adapted, customized forms of publicly available KEGG pathway maps, referenced in Table S3.

**Figure S6.** Pathway activity supporting vitamin B5 (Pantothenate) biosynthesis. Blue circle: conventional vitamin product. Red circle: active vitamin form. This content represents adapted, customized forms of publicly available KEGG pathway maps, referenced in Table S3.

**Figure S7.** Pathway activity supporting vitamin B6 (Pyridoxine) biosynthesis. Blue circle: conventional vitamin product. Red circle: active vitamin form. This content represents adapted, customized forms of publicly available KEGG pathway maps, referenced in Table S3.

**Figure S8.** Pathway activity supporting vitamin B7 (Biotin) biosynthesis. As the conventional vitamin is the active form in this case, Biotin is indicated by a red circle. This content represents adapted, customized forms of publicly available KEGG pathway maps, referenced in Table S3.

**Figure S9.** Pathway activity supporting vitamin B9 (Folate) biosynthesis. Blue circle: conventional vitamin product. Red circle: active vitamin form. This content represents adapted, customized forms of publicly available KEGG pathway maps, referenced in Table S3.

**Figure S10.** Pathway activity supporting vitamin B12 (Cobalamin) biosynthesis. Blue circle: conventional vitamin products. Red circle: active vitamin form. This content represents adapted, customized forms of publicly available KEGG pathway maps, referenced in Table S3.

**Figure S11.** Pathway activity supporting vitamin C (Ascorbate) biosynthesis. As the conventional vitamin is the active form in this case, Ascorbate is indicated by a red circle. This content represents adapted, customized forms of publicly available KEGG pathway maps, referenced in Table S3.

**Figure S12.** Pathway activity supporting vitamin K biosynthesis. As the conventional vitamins Phylloquinone (K1) and Menaquinone (K2) are the active forms in this case, both are indicated by red circles. This content represents adapted, customized forms of publicly available KEGG pathway maps, referenced in Table S3.

**Figure S13.** Predicted capacity of endosymbionts and non-symbiont relatives to synthesize active vitamin forms according to pathway map analysis. Red: pathways predicted to be “complete”. Orange: Pathways that were “assumed complete”. Cyan: “partial pathways”. Blue: “no capacity” for biosynthesis. A) Predicted capacity of the endosymbionts to synthesize each active vitamin form. B) Predicted capacity of the non-symbionts to synthesize each active vitamin form.

**Figure S14.** Testing for relationships between endosymbiont-host relationships and synthetic capacity of vitamin pathways. Grey patterns correspond to endosymbiont taxa listed in key. Red: pathways predicted to be “complete”. Orange: Pathways that were “assumed complete”. Cyan: “partial pathways”. Blue: “no capacity” for biosynthesis. A) Endosymbiont taxonomic profiles associated with each arthropod host order. B) Overall endosymbiont capacity for synthesizing active vitamin forms across host orders. C) Vitamin pathway capacity associated with primary and secondary endosymbionts overall (left) and in association with specific endosymbiont taxa (right).

**Figure S15**. Identification of predicted enzymes in each organism by the DataMiner application. Grey boxes represent data lists. White boxes describe stepwise analytical functions of the software.

**SUPPLEMENTARY TABLE LEGENDS**

**Table S1.** Information about the organisms discussed in this study. The taxonomic order of the organisms reflects outcomes reported by NCBI Taxonomy Tree.

**Table S2**. Vitamin-interacting proteins used to test for overlap of KEGG and NCBI Microbial BLAST annotations.

**Table S3.** KEGG pathway maps analyzed in association with production of each vitamin.

**Table S4.** Identification of homologs for the substrate-ambiguous enzymes IlvC and GapA. Table shows direct readouts from the Excel version of DataMiner used in this study. “1” indicates that a homolog of the enzyme was detected, whereas “0” indicates that no homolog was identified.

**Table S5.** Endosymbiont capacity for de novo biosynthesis of conventional vitamins. Red indicates that the pathways are “complete” up to the end-points assessed. Orange indicates pathways that were “assumed complete.” Cyan indicates “partial pathways.” Blue indicates “no capacity” for synthesis. Asterisk: Prediction includes contribution of a substitute enzyme.

**Table S6.** Endosymbiont capacity for de novo biosynthesis of active vitamin forms. Red indicates that the pathways are “complete” up to the end-points assessed. Orange indicates pathways that were “assumed complete.” Cyan indicates “partial pathways.” Blue indicates “no capacity” for synthesis. Asterisk: Prediction includes contribution of a substitute enzyme.

**Table S7.** Non-symbiont capacity for de novo biosynthesis of conventional vitamins. Red indicates that the pathways are “complete” up to the end-points assessed. Orange indicates pathways that were “assumed complete.” Cyan indicates “partial pathways.” Blue indicates “no capacity” for synthesis. Asterisk: Prediction includes contribution of a substitute enzyme. Double asterisk: Pathway has been confirmed as functional in vitro.

**Table S8.** P-values from various chi square tests. The table reports significant differences between endosymbiont taxonomic groups in the context of de novo vitamin biosynthesis, de novo biosynthesis of active vitamin forms, the vitamin conversion index, and the vitamin utility index.

**Table S9.** Non-symbiont capacity for de novo biosynthesis of active vitamin forms. Red indicates that the pathways are “complete” up to the end-points assessed. Orange indicates pathways that were “assumed complete.” Cyan indicates “partial pathways.” Blue indicates “no capacity” for synthesis. Asterisk: Prediction includes contribution of a substitute enzyme. Double asterisk: Pathway has been confirmed as functional in vitro.

**Table S10.** Possible alternate functions of vitamin biosynthesis pathways. Pathway predictions were defined as per the criteria outlined in Figure 1. Red indicates that pathways predicted as “complete” up to the end-points assessed. Cyan indicates “partial pathway” predictions. Blue indicates a prediction of “no capacity” for synthesis. Purple indicates predicted last-step conversion events specifically associated with vitamin B12.

**Table S11.** Data supporting vitamin conversion index predictions for all organisms analyzed. Red: Organism has one or more predicted homologs of enzymes that drive last-step conversion of intermediates into active vitamin forms. Blue: Organism had no predicted homologs of the related enzymes. The number of enzyme homologs corresponding to each case is directly displayed in the table.

**Table S12.** Data supporting vitamin utility index predictions for all organisms analyzed. Red: Organism has one or more predicted homologs of enzymes that require active vitamin forms as cofactors, or bind directly to the vitamins with unknown functional consequences. Blue: Organism had no predicted homologs of the related enzymes. The number of enzyme homologs corresponding to each case is directly displayed in the table.

**Table S13.** Vitamin dependency index per organism. This table summarizes comparisons of Table S12 to Table S14. Light grey: cases where last-step conversion of an intermediate into an active vitamin form was predicted, but no utility for the product was apparent. Medium grey: cases where capacity was predicted for both vitamin conversion and vitamin utility by the organism. Dark grey: cases where no capacity for vitamin conversion was detected, though utility of the active vitamin product was predicted

**SUPPLEMENTARY LEGENDS FOR ADDITIONAL FILES**

**File S1.** Full reference descriptions for the citations provided in in Table S1.

**File S2.** Source code for the DataMiner program.

**File S3.** Further source code for the DataMiner program.

**File S4.** The DataMiner software application. The software cross-references lists of enzymes with the endosymbionts and non-symbionts studied here, to report presence or absence of enzyme homologs in each organism. DataMiner accepts information from .txt files that contain lists of enzymes in terms of EC numbers (i.e. “3.1.3.9”), with one enzyme listed per line. After DataMiner finishes determining the presence/absence of enzyme homologs, the program deposits a .txt file containing space-delimited results on the desktop.

**File S5.** An Excel-based version of DataMiner. This was used to look up organisms encoding homologs of single enzymes, one enzyme at a time. After pulling up an enzyme entry in KEGG, the “genes” field was expanded to show all. The entire dataset from the “genes” field was transferred into columns A and B of the spreadsheet. Preset formulas in the spreadsheet textmine column A for matches to the organism abbreviations listed in column D. Matches are reported as “1” and indicated red, whereas absence of matches reported as a “0” and indicated blue in column E.

**File S6.** Lists of enzymes that convert intermediates into each active vitamin form. This file shows combined readouts from the java and excel version of DataMiner used in this study. “1” indicates that a homolog of the enzyme was detected. “0” indicates that no homolog was identified.

**File S7.** Lists of enzymes that utilize active vitamin forms. This file shows combined readouts from the java and excel version of DataMiner used in this study. “1” indicates that a homolog of the enzyme was detected. “0” indicates that no homolog was identified.
